# An R2R3-type MYB transcription factor, GmMYB29, regulates isoflavone biosynthesis in soybean

**DOI:** 10.1371/journal.pgen.1006770

**Published:** 2017-05-10

**Authors:** Shanshan Chu, Jiao Wang, Ying Zhu, Shulin Liu, Xiaoqiong Zhou, Huairen Zhang, Chun-e Wang, Wenming Yang, Zhixi Tian, Hao Cheng, Deyue Yu

**Affiliations:** 1 National Center for Soybean Improvement, National Key Laboratory of Crop Genetics and Germplasm Enhancement, Nanjing Agricultural University, Nanjing, Jiangsu, China; 2 Department of Agronomy, Henan Agricultural University, Zhengzhou, Henan, China; 3 State Key Laboratory of Plant Cell and Chromosome Engineering, Institute of Genetics and Developmental Biology, Chinese Academy of Sciences, Beijing, China; 4 College of Pharmacy and Life Science, Jiujiang University, Jiujiang, Jiangxi, China; University of Georgia, UNITED STATES

## Abstract

Isoflavones comprise a group of secondary metabolites produced almost exclusively by plants in the legume family, including soybean [*Glycine max* (L.) Merr.]. They play vital roles in plant defense and have many beneficial effects on human health. Isoflavone content is a complex quantitative trait controlled by multiple genes, and the genetic mechanisms underlying isoflavone biosynthesis remain largely unknown. Via a genome-wide association study (GWAS), we identified 28 single nucleotide polymorphisms (SNPs) that are significantly associated with isoflavone concentrations in soybean. One of these 28 SNPs was located in the 5’-untranslated region (5’-UTR) of an R2R3-type MYB transcription factor, *GmMYB29*, and this gene was thus selected as a candidate gene for further analyses. A subcellular localization study confirmed that *GmMYB29* was located in the nucleus. Transient reporter gene assays demonstrated that GmMYB29 activated the *IFS2* (isoflavone synthase 2) and *CHS8* (chalcone synthase 8) gene promoters. Overexpression and RNAi-mediated silencing of *GmMYB29* in soybean hairy roots resulted in increased and decreased isoflavone content, respectively. Moreover, a candidate-gene association analysis revealed that 11 natural *GmMYB29* polymorphisms were significantly associated with isoflavone contents, and regulation of *GmMYB29* expression could partially contribute to the observed phenotypic variation. Taken together, these results provide important genetic insights into the molecular mechanisms underlying isoflavone biosynthesis in soybean.

## Author summary

Isoflavones are bioactive substances with various benefits, and increasing isoflavone content is one of the major aims of soybean quality improvement. Isoflavone biosynthesis is regulated by multiple genes and complex metabolic networks. The modification of certain structural genes in the isoflavone pathway by genetic engineering has been unable to significantly improve isoflavone content. Thus, the identification and application of transcription factors specific to the isoflavone pathway may effectively resolve this problem. Here, a genome-wide association study (GWAS) was used to identify an R2R3-type MYB transcription factor, *GmMYB29*, associated with soybean isoflavone contents. Transient expression and plant transformation results confirmed that *GmMYB29* positively regulates isoflavone biosynthesis in soybean. A candidate-gene association analysis identified 11 probable causative *GmMYB29* polymorphisms. The identification and functional characterization of *GmMYB29* not only improves our understanding of the genetic molecular mechanisms underlying isoflavone synthesis but also provides a direct target for both genetic engineering and selection for the improvement of isoflavone content in soybean.

## Introduction

Isoflavones are a group of secondary metabolites predominantly distributed in leguminous plants, including soybean [*Glycine max* (L.) Merr.] [1]. In plants, isoflavones play important roles in microbial interactions, functioning as phytoalexins to protect plants from pathogen infection [2, 3]. They also act as signal molecules in the formation of nitrogen-fixing root nodules in leguminous plants [4]. For humans, isoflavones have health benefits in the prevention of several diseases, such as cancer [5], cardiovascular disease [6], and climacteric syndrome [7], which are associated with their phytoestrogenic and antioxidant properties [8]. However, isoflavones are undesirable in soy-based infant formulas [9]. In soybean breeding, an improved understanding of the mechanism of isoflavone biosynthesis would be of great value, as it may allow the manipulation of isoflavone biosynthesis and the production of cultivars that can meet various needs.

In soybean, there are three core isoflavone aglycones: daidzein, genistein, and glycitein [10]. They are synthesized via the general phenylpropanoid pathway that exists in all higher plant species and a branch of the isoflavonoid biosynthesis pathway specific to leguminous plants [11]. Isoflavone biosynthesis begins with the deamination of phenylalanine by phenylalanine ammonia lyase (PAL). After steps catalyzed by a series of enzymes, the critical branch point enzymes chalcone synthase (CHS) and isoflavone synthase (IFS) lead substrates to the isoflavone synthesis branch and finally generate isoflavones and their derivatives [12]. In addition to isoflavone biosynthesis, the phenylpropanoid pathway is also involved in the synthesis of lignins, stilbene, phlobaphenes, proanthocyanidins and anthocyanins via specific branches. Hence, the biosynthesis of isoflavones involves an intricate network reconciling many competing branch pathways. Thus, the modulation of a single gene does not necessarily alter the metabolic flux to target branch pathways [12–14].

The isoflavone biosynthesis pathway is complex, and functional differentiation is found in the isoflavone synthesis-related gene families due to two recent whole-genome duplication events: a soybean-lineage-specific duplication 13 million years ago and an early-legume duplication 59 million years ago [15, 16]. Therefore, researchers have focused on the discovery and application of isoflavone regulation-related transcription factors (TFs) instead of the manipulation of a single gene. Various TFs have been identified to regulate the biosynthesis of phenylpropane substances in higher plants, such as MYB, bZIP, WRKY, MADS box and WD40. Some MYB TFs involved in the regulation of the isoflavonoid biosynthesis pathway have been identified in soybean. For example, the R1-type MYB TF *GmMYB176* has been shown to affect isoflavonoid synthesis by regulating *CHS8* gene expression [1]. The R2R3-type MYB TFs *GmMYB39* and *GmMYB100* have been reported to negatively regulate isoflavonoid biosynthesis by suppressing the expression of structural biosynthesis genes [17, 18]. The soybean genome contains 4343 putative transcription factors, which account for 6.5% of the total predicted genes [19]. It is therefore challenging to discover and identify the key isoflavone regulation-related transcription factors at the genomic level.

Isoflavone content is a complex quantitative trait controlled by multiple genes and affected by both genetic and environmental factors. The primary mapping method for isoflavone-related quantitative trait loci (QTLs) is linkage analysis based on family lines, which is a classical method used to investigate complicated quantitative traits [19]. Previous studies have identified many QTLs controlling the biosynthesis of isoflavones in soybean seeds [20–25]. However, no isoflavone synthesis-related QTLs have been cloned due to limited allelic variation between recombinant inbred population parents. The application of genome-wide association study (GWAS), a more accurate method than linkage analysis, could enhance the power of functional gene identification [26, 27].

The development of the large genome-wide NJAU 355K SoySNP array in our previous study provides a useful tool facilitating GWAS in soybean [28]. In this study, we used this array to perform a GWAS for isoflavone contents and revealed a number of potential loci controlling isoflavone biosynthesis in soybean. We then demonstrated that one candidate gene, an R2R3-type MYB TF designated *GmMYB29*, played an important role in the regulation of isoflavone biosynthesis in soybean. Transcription analyses revealed a close correlation between the expression of *GmMYB29* and *IFS2* under normal and stressed conditions as well as between the expression of *GmMYB29* and the accumulation of isoflavones. Transient reporter gene assays and overexpression of *GmMYB29* in soybean hairy roots also strongly supported its key roles in the regulation of *IFS2* and *CHS8* expression and the isoflavone accumulation. Additionally, by combining a *GmMYB29*-based association analysis with an analysis of *GmMYB29* expression in seed samples of 30 natural soybean varieties, we confirmed the positive regulatory role of *GmMYB29* in isoflavone biosynthesis.

## Results

### Isoflavone contents in soybean seeds display significant variation

To determine the range of variation of isoflavone contents in soybean, the total isoflavone contents (TIC), daidzein contents (DAC), genistein contents (GEC) and glycitein contents (GLC) in soybean seeds were determined using 196 soybean accessions. To address the potential environmental influence, the soybean accessions were grown in two locations: Nanjing and Nantong (designed as two environments) (Table 1). A broad variation in isoflavone contents was observed in the population. For example, the GLC varied from 10.36 μg g^-1^ to 1794.00 μg g^-1^ in Nanjing. The average TIC, DAC, GEC and GLC was 5445.74 μg g^-1^, 3596.17 μg g^-1^, 946.73 μg g^-1^, and 423.85 μg g^-1^, respectively. The isoflavone contents showed continuous variation and normal distribution (S1 Fig), with skew and kurtosis less than one in the different environments. An analysis of variance (ANOVA) revealed that genotype and the genotype-by-environment interaction significantly influenced the major isoflavone contents (*P* < 0.001). This result supported the idea that isoflavone content is a complex trait controlled by multiple factors. However, the broad-sense heritability (*h*^*2*^) values of TIC, DAC, GEC and GLC were 74.1%, 76.3%, 67.8% and 83.8%, respectively, indicating that isoflavone content was primarily affected by genetic factors.

**Table 1:** Descriptive statistics, ANOVA results and broad-sense heritability (*h*^*2*^) of major isoflavone components of soybean across two environments.

Trait	Env.^a^	Mean	SD	Min	Max	Skew	Kurtosis	G^b^	E^c^	G×E^d^	*h*^2^(%)^e^
TIC	NJ	5319.69	2063.26	970.54	11594.82	0.34	0.12	***	ns	***	74.10
	NT	5571.79	2283.30	890.40	12676.28	0.17	-0.19				
DAC	NJ	3540.36	1627.06	250.19	8732.97	0.27	0.13	***	ns	***	76.30
	NT	3651.99	1566.35	291.60	9263.92	0.40	0.26				
GEC	NJ	1245.25	477.47	277.18	2893.17	1.00	1.30	***	**	***	67.80
	NT	648.20	811.16	237.72	3690.72	0.59	-0.29				
GLC	NJ	513.67	376.61	10.36	1794.00	0.36	-0.10	***	***	***	83.80
	NT	334.03	341.91	9.84	1496.92	0.88	-0.30				

TIC: total isoflavone contents; DAC: daidzein contents; GEC: genistein contents; GLC: glycitein contents; NJ: Nanjing; NT: Nantong; ns: not significant.

** significant at 0.01 probability levels;

*** significant at 0.001 probability levels;

^a^: environment;

^b^: genotype;

^c^: environment;

^d^: genotype × environment;

^e^: broad-sense heritability.

### GWAS identifies candidate genes for isoflavone biosynthesis in soybean

**Fig 1:**
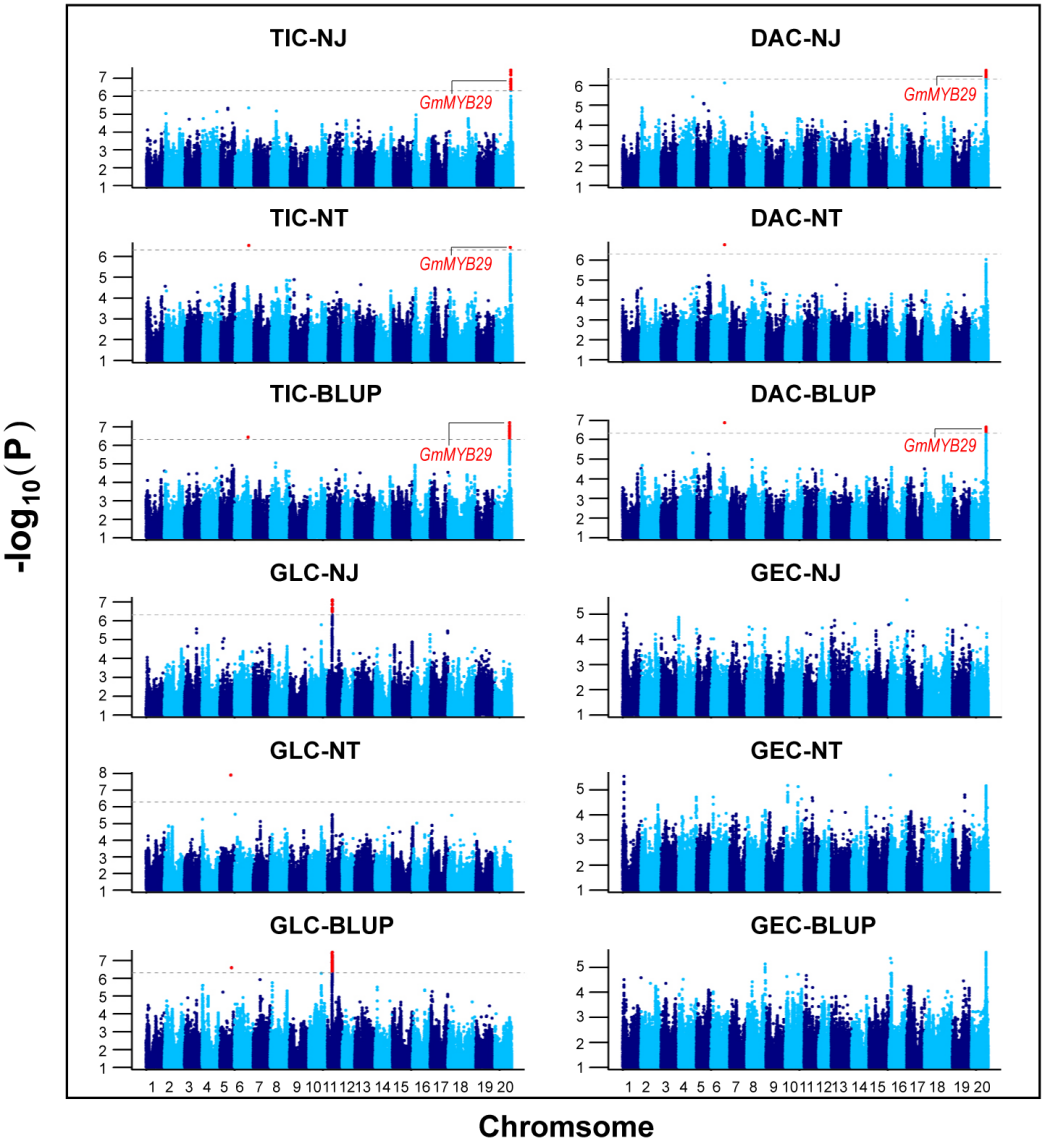
Manhattan plots of the GWAS for total isoflavone contents (TIC), daidzein contents (DAC), glycitein contents (GLC), and genistein contents (GEC) in the NJ environment, the NT environment and the BLUP data set across these two environments.

To identify the loci associated with isoflavone contents, GWAS was conducted using TIC, DAC, GEC, GLC and 207,608 SNPs with a minor allele frequency (MAF) > 0.05. These SNPs were obtained from the genotyping results of the 196 soybean accessions acquired using the NJAU 355K SoySNP array [28]. Twenty-eight SNPs significantly associated with the major isoflavone components were not only detected in the NJ or NT environment but also repetitively detected in the best linear unbiased prediction (BLUP) data set under a threshold of *P* < 4.82×10^−6^. These SNPs were considered as potentially reliable SNPs for further analysis (Table 2 and Fig 1). Additionally, 22 significant SNPs detected only once in the NJ environment, NT environment or the BLUP data set are presented in S1 Table. The 28 significant SNPs were located on chromosomes 5, 6, 11 and 20 and assembled into clusters on chromosomes 11 and 20. Among the significant SNPs, 17, 10, and 11 SNPs were associated with TIC, DAC, and GLC, respectively. Notably, the 10 SNPs significantly associated with DAC were overlapped with those associated with TIC. Unfortunately, no detected SNPs were significantly associated with GEC. The phenotypic variation explained by each of these significant 28 SNPs ranged from 10.20% to 14.98%, suggesting that major QTLs for isoflavone contents may exist.

**Table 2:** Details of SNPs significantly associated with major isoflavone components of soybean.

Marker	Chr.^a^	Position	Trait	*P* value^b^			*R*^*2*^(%)^f^	Transcription factor gene
				NJ^c^	NT^d^	BLUP^e^		
AX-93722354	5	36904003	GLC	ns	1.24E-07	2.55E-06	11.70–14.98	*Glyma05g31800*(*WRKY*), *Glyma05g31910*(*WRKY*)
AX-93734476	6	41283321	TIC	ns	3.02E-06	3.66E-06	10.43–10.98	*Glyma06g38340*(*MYB*), *Glyma06g38410*(*NAC*), *Glyma06g38440*(*NAC*)
			DAC	ns	1.70E-06	1.44E-06	11.59–11.64	
AX-93788682	11	8247884	GLC	3.27E-06	ns	1.66E-06	11.39–12.16	*Glyma11g11450*(*MYB*), *Glyma11g11540*(*bZIP*)
AX-93788683	11	8248245	GLC	3.27E-06	ns	1.66E-06	11.39–12.16	
AX-94286338	11	8252466	GLC	2.34E-06	ns	9.91E-07	11.75–12.72	
AX-93936943	11	8254189	GLC	1.35E-06	ns	6.57E-07	12.34–13.17	
AX-93788688	11	8262066	GLC	2.70E-06	ns	3.44E-06	11.38–11.60	*Glyma11g11570*(*MYB*)
AX-93788689	11	8262730	GLC	8.03E-07	ns	1.37E-06	12.37–12.90	
AX-93788692	11	8268689	GLC	9.14E-07	ns	1.67E-06	12.16–12.76	
AX-94085385	11	8269763	GLC	1.31E-06	ns	2.37E-06	11.78–12.37	
AX-93788694	11	8271460	GLC	9.19E-07	ns	1.52E-06	12.25–12.76	
AX-94085391	11	8284464	GLC	1.35E-06	ns	2.70E-06	11.64–12.34	
AX-94292181	20	43281039	TIC	3.10E-06	ns	3.26E-06	10.51–10.55	
AX-94208339	20	43283579	TIC	2.09E-06	ns	2.26E-06	10.90–10.91	
			DAC	4.01E-06	ns	ns	10.72	
AX-93910368	20	43285450	TIC	2.19E-06	ns	2.81E-06	10.70–10.85	
AX-93661412	20	43285529	TIC	1.88E-06	ns	1.86E-06	11.00–11.10	
AX-94208344	20	43294213	TIC	1.50E-06	ns	2.34E-06	10.88–11.22	
			DAC	3.56E-06	ns	ns	10.84	
AX-94208372	20	43428004	TIC	1.44E-06	ns	1.31E-06	11.27–11.46	
			DAC	4.35E-06	ns	4.47E-06	10.49–10.64	
AX-93661429	20	43452456	TIC	2.18E-06	ns	1.85E-06	10.86–11.11	
AX-93910415	20	43453176	TIC	1.61E-06	ns	1.71E-06	11.15–11.19	
AX-93910416	20	43458721	TIC	1.14E-06	3.72E-06	6.15E-07	10.76–12.22	***Glyma20g35180*(*MYB*)**
			DAC	4.57E-06	ns	2.85E-06	10.59–10.95	
AX-93910417	20	43460939	TIC	6.10E-07	ns	9.27E-07	11.80–12.12	
			DAC	2.74E-06	ns	3.67E-06	10.69–11.11	
AX-93661433	20	43465595	TIC	2.19E-06	ns	3.08E-06	10.60–10.85	
AX-94292195	20	43485102	TIC	4.30E-07	ns	8.55E-07	11.88–12.47	
			DAC		ns	2.42E-06	11.11–11.38	
AX-93910432	20	43490475	TIC	4.30E-07	ns	8.55E-07	11.88–12.47	
			DAC	2.11E-06	ns	2.42E-06	11.11–11.38	
AX-93910435	20	43497182	TIC	6.39E-07	ns	1.35E-06	11.42–12.07	
			DAC	2.11E-06	ns	3.41E-06	10.77–10.92	
AX-94208409	20	43502141	TIC	3.47E-07	ns	1.00E-06	11.72–12.69	*Glyma20g35280*(*AUX/IAA*)
			DAC	1.80E-06	ns	2.64E-06	11.03–11.54	
AX-93957635	20	43517749	TIC	4.25E-06	ns	4.30E-06	10.20–10.28	

Significant SNPs detected at least twice in the BLUP data set, NJ environment or NT environment are shown in this table; GLC: glycitein contents; TIC: total isoflavone contents; DAC: daidzein contents; bold type indicates that the SNP marker is located in the region of the gene.

^a^: chromosome;

^b^: significant at *P* < 4.82×10^−6^;

^c^: Nanjing;

^d^: Nantong;

^e^: BLUP data set across the two environments;

^f^: percentage of phenotypic variation explained by the SNP.

Based on the linkage disequilibrium (LD) decay calculated previously, the genes within 130 kb flanking the significant SNPs were selected [28]. Among these genes, no known genes in the isoflavone biosynthesis pathway were identified. Therefore, it was speculated that there could be novel genes related to isoflavone biosynthesis or regulation in these loci. Consistently, numerous TF-encoding genes, including MYB, NAC, bZIP, and WRKY were identified (Table 2). These TF-encoding genes could function in the regulation of isoflavone biosynthesis.

Among the 28 significant SNPs, there was only one SNP detected in NJ, NT and the BLUP data set. Notably, this SNP (AX-93910416) was detected within the 5’-untranslated region (5’-UTR) of *Glyma20g35180* (*GmMYB29*). Interestingly, the homologous gene *LjMYB14* has been characterized as a TF regulating isoflavonoid biosynthesis in Lotus (*Lotus japonicas*) [29]. These results suggested that *Glyma20g35180* could be a candidate gene controlling isoflavone biosynthesis in soybean.

### *GmMYB29* encodes an R2R3-type MYB transcription factor

The full-length open reading frame (ORF) of *GmMYB29* was 819 bp and encoded a protein of 272 amino acid residues with a calculated mass of 31.15 kDa and a pI of 5.77. The GmMYB29 protein was predicted to belong to the R2R3-type MYB subfamily. A multiple alignment of GmMYB29 with R2R3-type MYB TFs known to regulate isoflavonoids or flavonoids from various plant species showed a high homology in the N-terminal MYB domain (S2 Fig). GmMYB29 was clustered with AtMYB13, AtMYB14, AtMYB15, NtMYB2, DcMYB1, LjMYB13, LjMYB14, LjMYB15, VvMYB14, and VvMYB15, and they shared a C-terminal conserved motif found in subgroup 2 of the R2R3-type MYB gene family in *Arabidopsis* (the SG2 motif, DxSFW-MxFWFD), which has previously been described as a stress response motif (S2 Fig) [30–32]. Consistent with this finding, in the phylogenetic tree, the proteins from various plants that were grouped in the same cluster with GmMYB29 have been reported to respond to biotic or abiotic stresses (S3 Fig) [30]. Notably, LjMYB13, LjMYB14 and LjMYB15, which regulate isoflavonoid biosynthesis in Lotus [29], were found to form a cluster with GmMYB29.

### GmMYB29 protein is localized in the nucleus

To determine the subcellular localization of the GmMYB29 protein, the *GmMYB29* cDNA was fused with green fluorescent protein (GFP) under the control of the CaMV 35S promoter. This construct was then transformed into *Arabidopsis* mesophyll protoplasts using polyethylene glycol (PEG) and into onion epidermal cells using a gene gun. Consistent with the putative function of TFs, the GmMYB29::GFP fusion protein was localized in the nucleus, while in cells transformed with a GFP control plasmid, fluorescence was detected in both the cytoplasm and the nucleus (S4 Fig).

### The expression levels of *GmMYB29* are associated with isoflavone content

**Fig 2:**
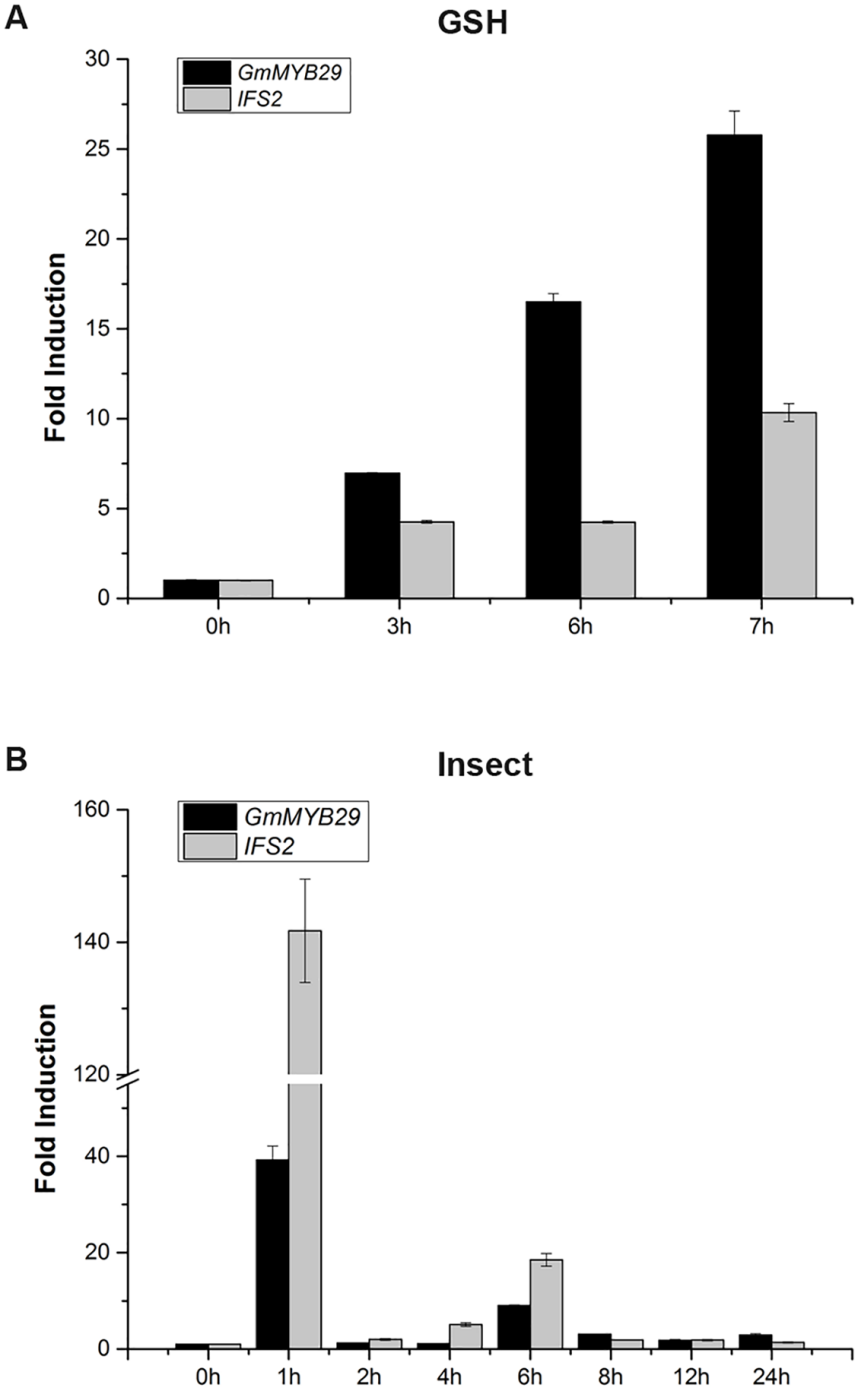
Expression of *GmMYB29* and *IFS2* in response to glutathione (GSH) and insect treatments. (A) Expression of *GmMYB29* and *IFS2* in response to GSH treatment. (B) Expression of *GmMYB29* and *IFS2* in response to insect treatment. The experiment was repeated twice with the same results. The results of one of these experiments are shown. Bars indicate the standard errors of three technical replicates.

Previous studies have revealed that glutathione (GSH) treatment could induce isoflavonoid production [33] and that biotic stress could influence isoflavone content [34]. To determine whether the expression of *GmMYB29* was induced by GSH and biotic stress and whether the expression pattern of *GmMYB29* was consistent with that of isoflavone synthase 2 (*IFS2*), a key gene in isoflavone biosynthesis, we examined the expression of *GmMYB29* and *IFS2* in soybean leaves treated with GSH and common cutworms (Fig 2). After 3 h of GSH treatment, *GmMYB29* and *IFS2* showed 7- and 4-fold higher expression in treated leaves than in the control samples, respectively. After 7 h of treatment, these two genes showed 26- and 10-fold increases in expression, respectively. After insect-mediated induction, both *GmMYB29* and *IFS2* displayed marked up-regulation at 1 h and 6 h. Therefore, both *GmMYB29* and *IFS2* showed induced expression by GSH elicitation and insect feeding, and they were co-expressed under these two stresses. These results indicated that *GmMYB29* and *IFS2* could be involved in similar or the same biological processes.

**Fig 3:**
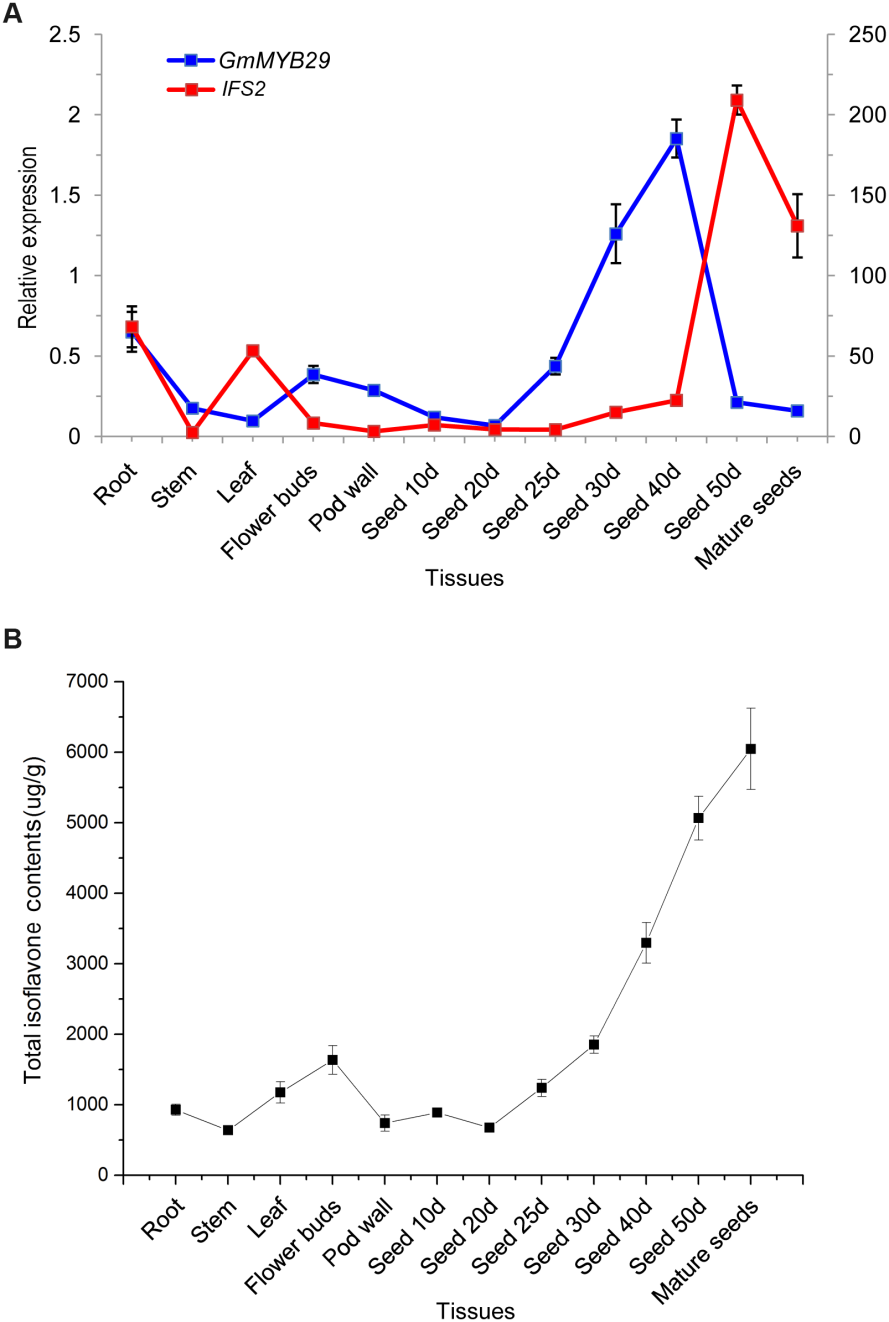
Expression of *GmMYB29* and *IFS2* and total isoflavone contents in different soybean tissues. (A) Expression of *GmMYB29* and *IFS2* in different soybean tissues. Bars represent the standard errors of three technical replicates for two biological replicates. (B) Accumulation pattern of total isoflavones in different soybean tissues. Bars represent the standard errors of three technical replicates for two biological replicates.

To further examine the correlation between *GmMYB29* and *IFS2* expression in different tissues and developmental stages of soybean, we investigated the expression patterns of *GmMYB29* and *IFS2* and the isoflavone content in different soybean tissues (Fig 3). The expression of *GmMYB29* was closely associated with that of *IFS2*. These two genes showed relatively higher expression in roots and seeds than in other tissues, and the expression noticeably increased with seed development. Notably, the expression of *GmMYB29* and *IFS2* was consistent with the isoflavone content in different tissues, suggesting that the expression of *GmMYB29* and *IFS2* is closely related with isoflavone accumulation in soybean.

### GmMYB29 activates the expression of *IFS2* and *CHS8*

**Fig 4:**
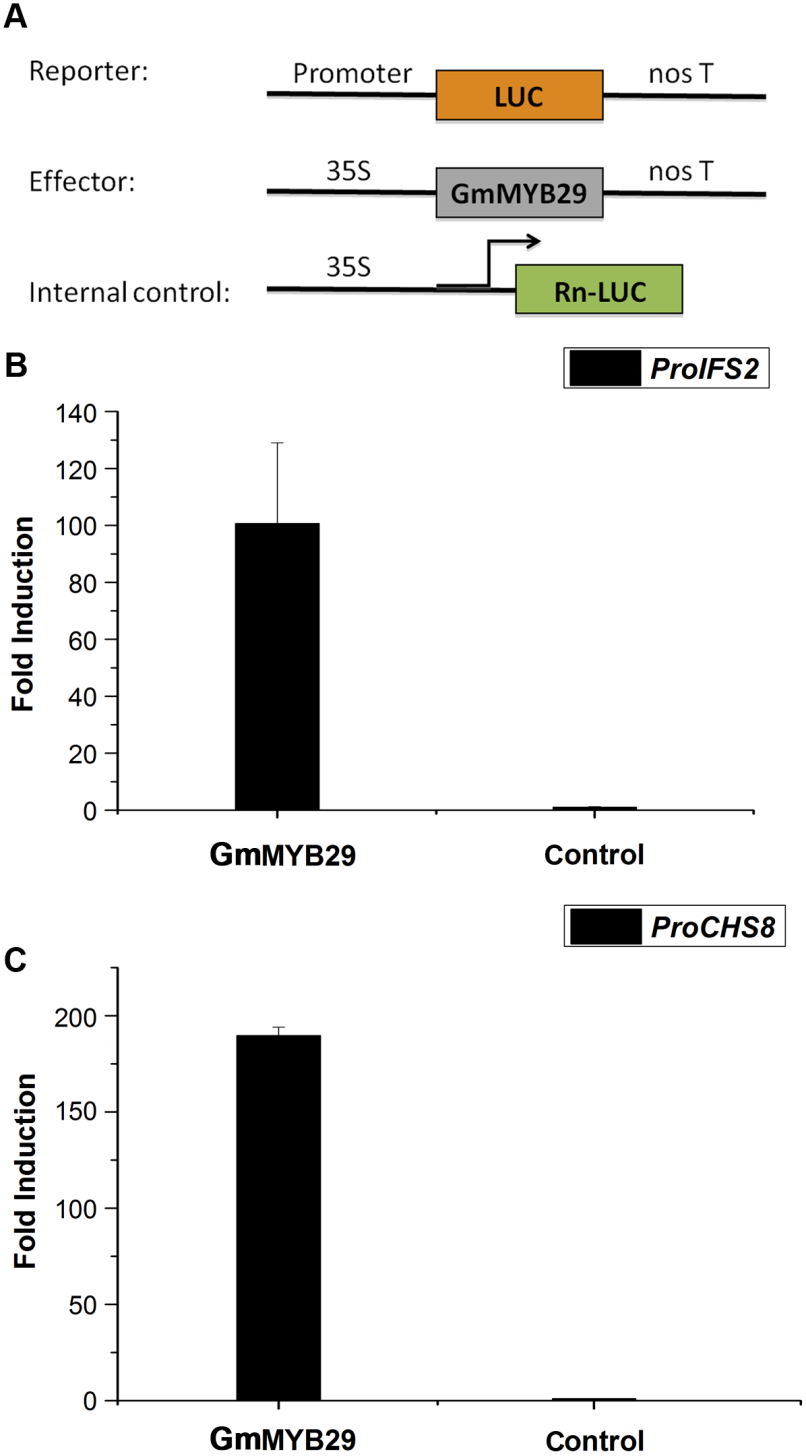
GmMYB29 induced the promoter activity of *IFS2* and *CHS8*, which are key structural isoflavone biosynthesis genes. (A) Schematic representation of the reporter, effector and internal control constructs used in the transient assays. (B) GmMYB29 induced the promoter activity of *IFS2*. GmMYB29 column represents relative LUC activity (Firefly/Renilla) of the *IFS2* promoter plus GmMYB29 factor relative to the control (without the GmMYB29 factor). The control contains the reporter and internal control plasmids. Bars indicate the standard errors of three technical replicates for three independent biological replicates. (C) GmMYB29 induced the promoter activity of *CHS8*. GmMYB29 column represents relative LUC activity (Firefly/Renilla) of the *CHS8* promoter plus GmMYB29 factor relative to the control (without the GmMYB29 factor). The control contains the reporter and internal control plasmids. Bars indicate the standard errors of three technical replicates for three independent biological replicates.

To examine whether GmMYB29 could regulate the expression of isoflavone biosynthesis-related genes, transient expression using *Arabidopsis* mesophyll protoplasts and a dual luciferase reporter gene assay was performed. The promoters of two critical genes (*IFS2* and *CHS8)* in the isoflavone biosynthesis pathway were amplified from 1790 bp and 1663 bp upstream of the start codons of *IFS2* and *CHS8*, respectively, to study the interaction between these promoters and GmMYB29. Several MYB binding elements and stress-related cis-regulatory elements were predicted in the *IFS2* and *CHS8* promoters using the PLACE database (http://www.dna.affrc.go.jp/htdocs/PLACE/) [35]. As shown in Fig 4, transient expression demonstrated that overexpression of *GmMYB29* increased the activity of both the *IFS2* and *CHS8* promoters by 100- and 200-fold, respectively, compared with the controls. These results suggested that GmMYB29 plays a critical role in the transcriptional regulation of key genes in the soybean isoflavone biosynthesis pathway.

To further identify the GmMYB29 recognition regions in the *IFS2* promoter, eight fragments (IFS2ΔP1-IFS2ΔP8) were generated by gradual 5’ deletions of the promoter, which were then used to drive luciferase (LUC) expression (S5 Fig). The reporters IFS2ΔP1-IFS2ΔP8_pro_:LUC and IFS2full_pro_:LUC were co-transfected into *Arabidopsis* protoplasts with 35S_pro_:*GmMYB29*, and the LUC activity was measured. The vectors containing IFS2ΔP1 and IFS2ΔP2 showed similar LUC activity to that containing IFS2full. However, the LUC activity dramatically decreased for IFS2ΔP3 and the further deletions. These results indicated that the 208 bp region between -885 and -1093 in the *IFS2* promoter contained the motif required for promoter activity. In this region, a cis-regulatory element, MYBCORE (containing the CNGTTR sequence), was predicted by PLACE as a MYB binding element, suggesting that GmMYB29 could bind the *IFS2* promoter and activate *IFS2* expression via the recognition of this element.

### Soybean hairy root transformation confirms the function of *GmMYB29*

**Fig 5:**
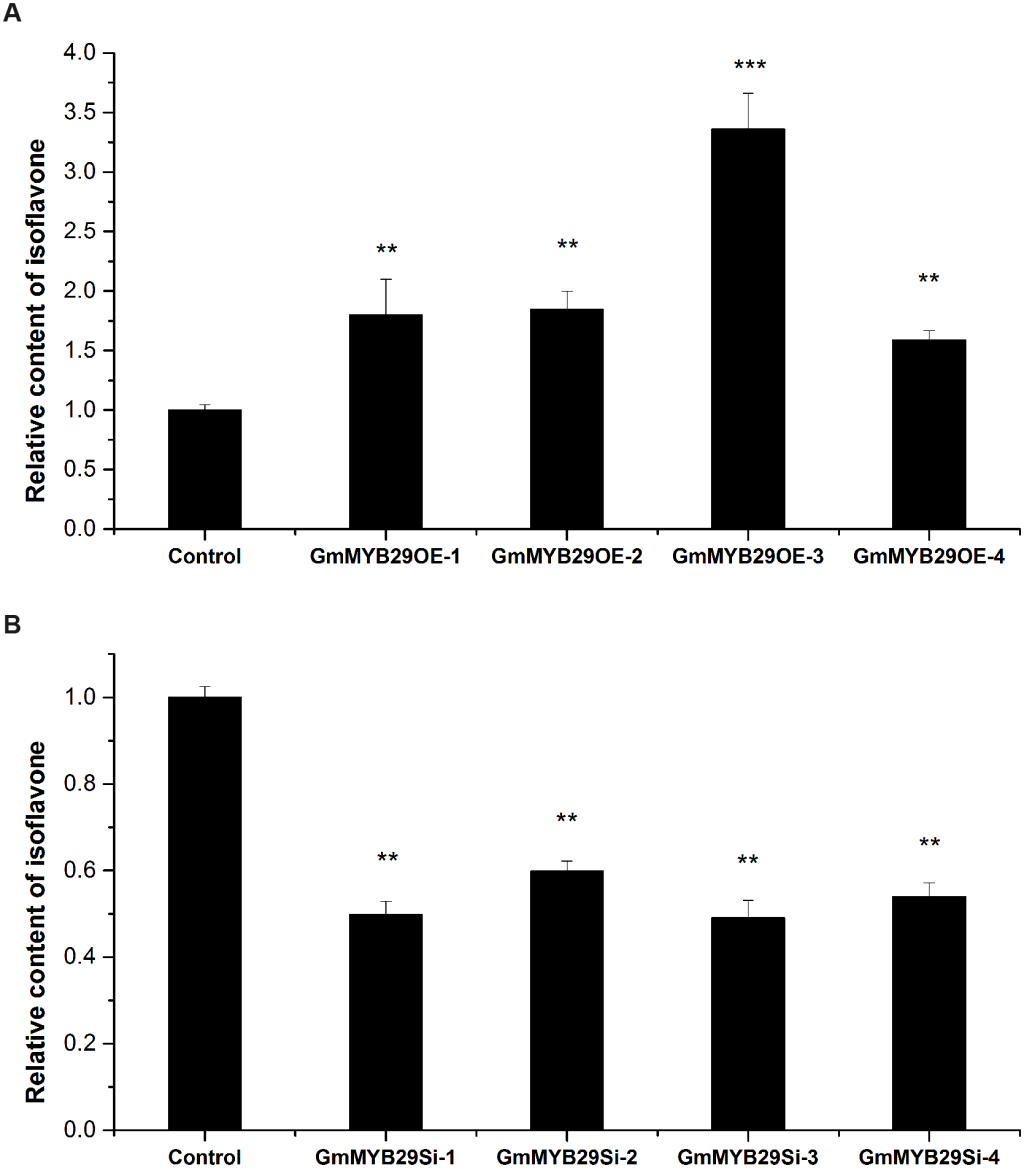
Isoflavone contents in *GmMYB29*-overexpressing and *GmMYB29*-silenced soybean hairy roots. (A) Overexpression of *GmMYB29* leads to increased isoflavone content in soybean hairy roots. GmMYB29OE1-4 represent four independent *GmMYB29*-overexpressing roots. The data for each of the four independent OE lines or control represent the means of three replicates with error bars indicating SE. (B) Silencing of *GmMYB29* leads to decreased isoflavone content in soybean hairy roots. GmMYB29Si1-4 represent four independent *GmMYB29*-silenced roots. The data for each of the four independent Si lines or control represent the means of three replicates with error bars indicating SE. ** significant at the 0.01 probability level; *** significant at the 0.001 probability level.

To determine the role of *GmMYB29* in isoflavone accumulation, overexpression and RNAi-mediated silencing of *GmMYB29* were performed using a soybean hairy root system (S6 Fig). The transgenic hairy roots were verified by PCR amplification, and the positive lines were selected to conduct further studies (S7 Fig). We performed quantitative RT-PCR to study the effect of overexpression and RNAi silencing on the transcription levels of *GmMYB29* and isoflavone biosynthesis-related genes, including *PAL*, cinnamate 4-hydroxylase (*C4H*), 4-coumarate coenzyme A ligase (*4CL*), *CHS8*, chalcone isomerase (*CHI*), chalcone reductase (*CHR*) and *IFS2*, in hairy roots obtained from several independent transgenic lines. The transcription level of *GmMYB29* was significantly increased by 14- to 47-fold in *GmMYB29*-overexpressing transgenic hairy roots (S8A Fig) and significantly reduced by 3- to 7-fold in transgenic hairy roots with RNAi-mediated *GmMYB29* silencing (S8B Fig). The *GmMYB29*-overexpressing transgenic roots also showed increased transcription levels of *PAL*, *4CL*, *CHS8*, *CHR*, and *IFS2*, but no significant change in *C4H* and *CHI* expression was observed between overexpressing and control roots (S9A Fig). Interestingly, the transcription levels of all the monitored isoflavone biosynthesis genes were markedly decreased in *GmMYB29*-silenced transgenic roots (S9B Fig). Furthermore, we measured the total isoflavone contents in *GmMYB29*-overexpressing and *GmMYB29*-silenced lines by high-performance liquid chromatography (HPLC). The isoflavone content increased by 1.6- to 3.3-fold in *GmMYB29*-overexpressing roots (Fig 5A) and decreased by 2-fold in the gene-silenced roots (Fig 5B) (*P* < 0.01).

### Polymorphisms in the *GmMYB29* gene are associated with isoflavone contents in soybeans

**Fig 6:**
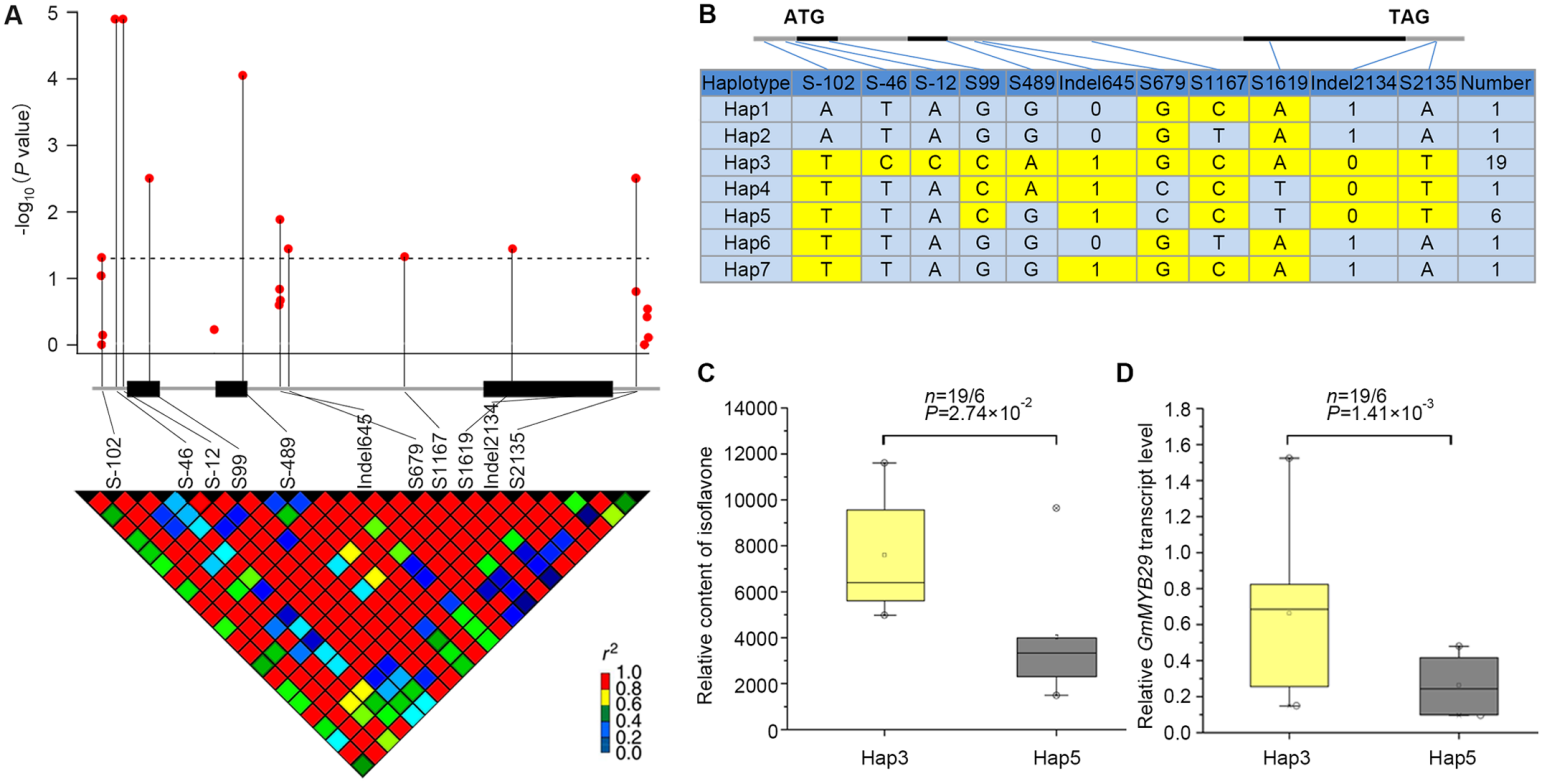
Natural variations in *GmMYB29* are significantly associated with total isoflavone contents in soybean. (A) *GmMYB29*-based association mapping and pairwise LD analysis. The red dots above the dashed horizontal line represent significant variants (*P* < 0.05), which are connected to the pairwise LD diagram with a solid line. (B) Haplotypes of *GmMYB29* among soybean natural variations. The haplotype is displayed as a linear combination of alleles (S-102, S-46, S-12, S99, S489, Indel645, S679, S1167 S1619, Indel2134 and S2135), and only the observed haplotypes are exhibited. The colored cells represent the favorable alleles. (C) Comparison of isoflavone contents between haplotypes Hap3 and Hap5. n denotes the genotype number of the two haplotypes. Statistical significance was detected by a two-tailed t-test. (D) Comparison of *GmMYB29* expression between haplotypes Hap3 and Hap5. n is the number of genotypes of the two haplotype groups. Statistical significance was determined by a two-tailed t-test.

To further investigate the association between the allelic variation of *GmMYB29* and isoflavone contents, we sequenced the *GmMYB29* gene in a subset of 30 soybean accessions, representing varieties with high, medium and low isoflavone contents. An approximately 2.4-kb genomic region, spanning the 5'- to 3'-UTR of *GmMYB29*, was analyzed. A total of 12 SNPs and 11 indels (insertions and deletions) were identified and filtered out for the subsequent association analyses (Fig 6A). The association study showed that 11 probable causative sites, including Site-102 (located 102 bp upstream from the translation start codon, S-102), S-46 and S-12 in the 5'-UTR, S99 in exon1, S489 in exon2, Indel645, S679 and S1167 in intron2, S1619 in exon3 and Indel2134 and S2135 in the 3'-UTR, were significantly associated with variations in the TIC (Table 3). S-12 (corresponding to SNP AX-93910416 in our GWAS results) and S-46 were significantly correlated with the TIC, both contributing to 49.99% of the phenotypic variation in the representative subset. A single-base transversion at S1619 resulted in an amino acid substitution of lysine to asparagine at amino acid position 133, which contributed to 14.91% of the variation in TIC.

**Table 3:** *GmMYB29* polymorphisms associated with total isoflavone contents.

Polymorphic site^a^	Allele^b^	*P* value^c^	*R*^*2*^ (%)^d^	Location	Amino acid change
S-102	T,A	4.74E-02	13.32	5' UTR	No
S-46	C,T	1.25E-05	49.99	5' UTR	No
S-12	C,A	1.25E-05	49.99	5' UTR	No
S99	C,G	3.10E-03	27.23	Exon1	No
S489	A,G	8.82E-05	42.79	Exon2	No
Indel645	0,1	1.27E-02	20.19	Intron2	No
S679	G,C	3.50E-02	14.91	Intron2	No
S1167	C,T	4.63E-02	13.44	Intron2	No
S1619	A,T	3.50E-02	14.91	Exon3	p.Lys133Asn
Indel2134	0,1	3.10E-03	27.23	3' UTR	No
S2135	T,A	3.10E-03	27.23	3' UTR	No

^a^: Only significant polymorphic sites are shown.

^b^: The favorable alleles are underlined (higher TIC).

^c^: Phenotypic data across two environments were used for the analysis.

^d^: percentage of phenotypic variation explained by the SNP.

Furthermore, based on the 11 significant variants with strong LDs (Fig 6A), the 30 soybean genotypes were classified into seven haplotype classes (Hap1-Hap7) (Fig 6B). Haplotype 3 (Hap3, n = 19) is the largest group, and Hap5 (n = 6) is the second largest group, whereas the other five haplotype classes are minor groups, each comprising one soybean accession. Statistically, Hap3 accessions had significantly higher TIC than Hap5 accessions (Fig 6C). Among the different sites between Hap3 and Hap5, the most significant variants are S-46 and S-12, which are located in the 5'-UTR. Considering that the expression of *GmMYB29* may cause phenotypic variation, we subsequently measured the expression of *GmMYB29* in seeds from these 30 soybean accessions. The expression of this gene was positively correlated with the isoflavone content (r = 0.63, *P* < 0.01) (S2 Table). Additionally, we observed that the Hap3 accessions exhibited higher *GmMYB29* expression than the Hap5 accessions (Fig 6D). Therefore, these data suggested that the expression of *GmMYB29* could at least partially explain the phenotypic variation in isoflavone content.

## Discussion

The isoflavone biosynthesis network is extremely complicated, and isoflavone accumulation is dependent on pathway enzymes and interactions among enzymes [36, 37]. However, modification of a single enzyme does not significantly alter isoflavone content [12]. Thus, the identification and application of specific transcription factors in the isoflavone pathway could be an effective method to resolve this problem. In this study, we successfully cloned a GWAS-identified transcription factor (*GmMYB29*) that was responsible for the isoflavone contents in soybean. We then combined expression analysis, transient expression analysis, soybean hairy root transformation, and candidate-gene association analyses to confirm that *GmMYB29* plays a positive regulatory role in soybean isoflavone biosynthesis. Our results reveal that an effective strategy for identifying the key QTL genes and provide a reference for cloning the rest of the isoflavone regulation-related loci in soybean as well as in other plants.

### GWAS is a powerful way to identify key genes associated with soybean isoflavone contents

Previously, a number of QTLs associated with soybean isoflavone-related traits have been identified by linkage mapping, but few of these have been cloned or functionally characterized, perhaps because of the QTL resolution, which is limited by lower density molecular markers [38]. GWAS with high-density markers can overcome this limitation and have recently been successfully applied in studies of *Arabidopsis thaliana*, rice and maize [39–41]. In the present study, using a diverse natural population genotyped with high-density markers (292,053 SNPs, one SNP/3.3 kb) and phenotyped in various environments, we identified an important TF related to soybean isoflavone biosynthesis, clarified its molecular mechanism and determined the favorable alleles/haplotypes.

Studies have shown that the selection of appropriate mapping populations genotyped with saturated markers is important for performing GWAS to identify complex QTLs [42]. The 196 accessions used in this study have been reported to identify QTLs associated with seed shape, phosphorus efficiency and yield, among other features [43–45], suggesting that this panel might contain diverse genetic variations in complex quantitative soybean traits. As expected, many genetic variations in TIC, DAC, GEC and GLC were observed in the association mapping population. In addition, DAC was always the highest, followed by GEC and then GLC across various environments, which was consistent with a previous report [25]. Although isoflavone content was affected by both the genotype and the interaction between genotype and environment, isoflavone content also maintained a high heritability (0.68–0.84), which agreed with recently reported results [22–25]. These studies reveal that the heritability of isoflavone content is high enough to be considered in breeding practices to genetically improve cultivars effectively.

GWAS based on high-density SNP markers can be used to finely map quantitative trait genes, even to the genes themselves. Recently, an 82-bp (MITE) insertion in the promoter region of a NAC gene (*ZmNAC111*) detected by a GWAS has been determined to be associated with maize drought tolerance [46]. In our study, a highly significant SNP, AX-93910416, was identified to be associated with soybean isoflavone contents across two environments (NJ and NT) and their BLUP. A strong LD was detected in the region around this SNP (S10 Fig), indicating the existence of artificial selection and a potential target gene responsible for phenotypes in this region. More importantly, this SNP was located in the 5’-UTR of the transcription factor *GmMYB29*. In addition, *GmMYB29* is homologous to *LjMYB14*, a transcription factor reported to regulate isoflavone biosynthesis in *Lotus corniculatus* [29], indicating that *GmMYB29* is possibly involved in isoflavone regulation.

### *GmMYB29* regulates isoflavone biosynthesis in soybean

It is known that similar and conserved protein functions are derived from conserved motifs from a common origin. Comparative genomic analyses have shown that GmMYB29 not only maintains the highly conserved R2R3 domain but also has a small amino acid motif in the C-terminal region. This small motif is SG2 (DxSFW-MxFWFD), which has been reported to be related to stress resistance in plants [30–32, 47, 48]. For example, AtMYB15, which contains an SG2 motif, reportedly can improve stress resistance by increasing the expression of genes related to ABA synthesis and the ABA signaling pathway in *Arabidopsis* plants exposed to drought and salinity [49, 50]. Similarly, our results showed that the expression of *GmMYB29* was significantly increased under abiotic and biotic stress. Interestingly, the expression pattern of *GmMYB29* was similar to that of *IFS2*, a key structural gene in the isoflavone biosynthesis pathway, which suggests that *GmMYB29* could be involved in the same regulation pathway as *IFS2* [29, 30]. Furthermore, the expression profile of *GmMYB29* determined by quantitative RT-PCR analysis in different tissues showed that *GmMYB29* was expressed in every isoflavone-accumulating tissue [51]. However, the expression of *GmMYB29* preceded *IFS2* in different developmental stages of soybean seeds. For instance, the highest expression levels of *GmMYB29* and *IFS2* occur on the 40^th^ and 50^th^ day after flowering, respectively. This is consistent with the hypothesis that the expression of regulators occurs in advance of their target genes [30]. These results indicate that *GmMYB29* may regulate isoflavone biosynthesis in soybean.

Transcription factors often act on the promoter region of their target genes and regulate their expression [52, 53]. As expected, we found that GmMYB29 can interact with the promoters of *IFS2* and *CHS8* and activate the expression of these two genes. Furthermore, co-transfection of promoter deletion fragments showed that a 208-bp fragment (from -885 bp to -1093 bp), which contains the MYB TF binding cis-acting element MYBCORE (CNGTTR), was necessary for the activation of IFS2_pro_:LUC, indicating the important role of this element in MYB recognition and gene transcriptional regulation. In addition, two other MYBCORE elements, located in the -1158 bp to -1790 bp and -1093 bp to -1158 bp regions of the *IFS2* promoter, respectively, were identified by cis-acting element prediction software. However, gradual deletions of these two elements (generating IFS2ΔP1 and IFS2ΔP2, respectively) showed no significant effects on LUC activity (S5 Fig). The IFS2ΔP3 construct, in which all of the MYBCORE elements were deleted, exhibited almost no LUC activity, confirming that the MYB elements are the key sites recognized by GmMYB29, thereby affecting *IFS2* transcription. The promoter sequences of other structural genes (*PAL1*, *C4H*, *4CL*, *CHS8*, *CHI*, *CHR* and *IFS1*) in the isoflavone pathway were also investigated, and various MYBCORE elements were identified. Thus, further experiments are required to confirm whether GmMYB29 directly interacts with other isoflavone pathway-related genes.

The isoflavone biosynthesis-related R2R3-type MYB TFs reported by previous studies have generally been negative regulators in soybean [17, 18]. For example, *GmMYB100* was found to inhibit isoflavonoid production by down-regulating the expression of *CHS*, *CHI* and *IFS* [17]. In addition to the negative regulators, the R1-type MYB TF *GmMYB176*, which could activate the promoter activity of *CHS8*, was also observed. RNAi-mediated silencing of *GmMYB176* in transgenic soybean hairy roots resulted in reduced levels of isoflavonoids. Unfortunately, overexpression of *GmMYB176* was not sufficient to increase *CHS8* transcription and isoflavonoid levels in hairy roots [1]. In our study, overexpression of *GmMYB29* increased the activity of *IFS2* and *CHS8* promoters; moreover, the isoflavone content was increased in *GmMYB29*-overexpressing hairy roots and decreased in *GmMYB29*-silenced hairy roots. These results imply that we identified a novel R2R3-type MYB TF, *GmMYB29*, which acts as a positive regulator to activate isoflavone production. Surprisingly, the level of isoflavone production in *GmMYB29*-overexpressing hairy roots was found to be only 3.3-fold higher at most than the control lines. One possible explanation for this observation may be the possible phytotoxic effect of isoflavonoid accumulation on the growth of hairy roots, as reflected in the relatively slow growth of *GmMYB29*-overexpressing transgenic hairy roots and the loss of several lines with high *GmMYB29* expression.

Soybean hairy roots overexpressing *GmMYB29* showed a marked increase in the expression of *PAL*, *4CL* and *CHS8* as well as *CHR* and *IFS2* (S9A Fig). This suggests that in addition to its role in the regulation of isoflavone biosynthesis, *GmMYB29* may also be involved in the regulation of upstream phenylpropanoid pathway genes to ensure the availability of substrates for isoflavone biosynthesis. It has been reported that a single TF could regulate multiple genes in the phenylpropanoid pathway and that expression of a single target gene in the pathway might be regulated by multiple TFs [54–58]. The transcriptional regulation of the anthocyanin and proanthocyanidin pathway genes is conducted by a complex in which R2R3-type MYB TFs, WD40 proteins, and bHLH proteins all interact to activate gene transcription [59–61]. Thus, we cannot exclude the possibility that there are other TFs that can activate the biosynthesis of isoflavones. Further characterization of other TFs identified in this research would provide deeper insight into the regulatory mechanisms underlying isoflavone biosynthesis.

In addition to transformation, the selection and accumulation of elite alleles of key genes functioning in isoflavone biosynthesis may be an effective strategy for soybean breeding. Similar investigations have been reported for maize, rice, soybean, and *Arabidopsis*, among others [46, 62–65]. For example, a sequence analysis of the drought tolerance gene *ZmVPP1* in 140 inbred maize lines identified a 366-bp insertion in the promoter, which was associated with maize drought tolerance and conferred drought-inducible expression of *ZmVPP1* in drought-tolerant accessions. Although some isoflavone biosynthesis-related TFs have been characterized [1, 17, 18], the polymorphism and haplotype analyses of these genes and the potential regulation mechanisms have not been reported. In this study, haplotype analysis showed that the *GmMYB29* gene can be found as seven haplotypes (Hap1-Hap7), and Hap3 had higher levels of isoflavone content and *GmMYB29* expression than the others, indicating that Hap3 might be significant to breeding soybeans with higher isoflavone content. Here, the 30 soybean accessions used to identify the favorable haplotype of *GmMYB29* were selected to represent soybeans with different levels of isoflavone content. However, as more than 20,000 soybean accessions have been preserved [66], other elite alleles of *GmMYB29* might be discovered using the stored soybean germplasms. The optimal haplotypes and alleles of this gene could therefore be detected by investigating the genetic differences in *GmMYB29* expression and transcriptional activity in additional soybean accessions. Taken together, these results could lead to the development of molecular markers for the breeding of soybeans with optimized isoflavone content.

## Materials and methods

### Plant materials

A natural population of 196 representative cultivated soybean (*Glycine max*) accessions with broad variations in isoflavone contents was selected from Wang *et al*. to develop the association mapping panel (S3 Table) [28]. These accessions (including 164 landraces, 24 improved accessions and 8 accessions with unknown evolution type) originated from all three ecological habitats of soybean in China. The seeds of each accession were provided by the Germplasm Storage of the Chinese National Center for Soybean Improvement (Nanjing Agricultural University, Nanjing, China). The trials were conducted in 2014 at two locations: Jiangpu Station of Nanjing Agricultural University in Nanjing (32°12'N, 118°37'E) (designated as environment NJ) and the Experimental Farm of Jiangsu Yanjiang Institute of Agricultural Sciences in Nantong (31°58'N, 120°53'E) (designated as environment NT). In each environment, 196 soybean accessions (corresponding to 196 plots) were planted in a randomized complete block design with three replicate blocks. Each accession was planted in four rows per plot, and each row was 200 cm long, with a row-spacing of 50 cm. The inter-plot spacing was also 50 cm. All field management requirements during the growing period, including watering, weed management, and fertilization, were performed similarly at both test locations. After maturity, four individuals from each plot were randomly screened for isoflavone content analysis.

### Isoflavone extraction and qualification

The extraction and determination of isoflavones was performed according to the protocol described by Sun *et al*. [67]. First, approximately 20 g of dried seeds from each accession was ground to a fine powder using a cyclone mill. Fifteen milligrams of this powder was added to a 2 mL tube containing 1.5 mL of 80% methanol. The mixture was spun for 30 s, subjected to ultrasound treatment (frequency 40 kHz, power 300 W) for one hour at 50°C, and rotated every ten minutes. After centrifugation at 12,000 rpm for 10 min, the supernatant was filtered using a YMC Duo-filter (YMC Co., Kyoto, Japan) with 0.22 μm pores. The sample was injected into a 2 mL Agilent auto sampler and stored at -20°C before use.

Samples were analyzed with a high-performance liquid chromatography (HPLC) system (Column: Zorbax SB-C18, 5 μm, 80 Å, 4.6 mm×150 mm) under the following conditions: solvent A was 0.1% aqueous acetic acid and solvent B was 100% methanol; the solvent system was 0–2 min 27% B (v/v), 2–3 min 27%-38% B, 3–10 min 38% B, 10–12 min 38%-39% B, 12–14 min 39% B, and 14–15 min 39%-27% B. The solvent flow rate was 2 mL/min, and the UV absorption was measured at 254 nm. The column temperature was set at 36°C, and the injection volume was 10 μL.

The identification and quantification of each isoflavone component was based on the standards of 12 isoflavone components provided by Sigma-Aldrich. The 12 isoflavone standards were daidzin (D), glycitin (GL), genistin (G), daidzein (DE), glycitein (GLE), genistein (GE), malonyldaidzin (MD), malonylglycitin (MGL), malonylgenistin (MG), acetyldaidzin (AD), acetylglycitin (AGL), and acetylgenistin (AG). Different concentration gradients (0, 5, 10, 20, 50, 100, 500, 1000 ng/sampler) of the 12 isoflavone standards in 80% methanol were produced. Twelve standard curves were generated to calculate the 12 kinds of isoflavone monomer content. The precise contents of these 12 isoflavone components per gram of dry seeds (μg g^-1^) were calculated with the formula described in detail by Sun *et al*. [67]. The total isoflavone contents (TIC) were calculated as the sum of the 12 isoflavone components. The contents of daidzein (DAC), genistein (GEC) and glycitein (GLC) were the sum of four corresponding components: malonyl glycosides, acetyl glucosides, β-glycosides and aglycones.

### Phenotypic data analysis

An analysis of variance (ANOVA) of all phenotypic data was performed using PROC GLM of SAS/STAT 9.2 (SAS Institute, 2002) with environment, replication within environment, genotype and genotype × environment as random effects. The ANOVA was based on the model *y*_*ijr*_ = *μ*+*G*_*i*_+*E*_*j*_+(*GE*)_*ij*_+*B*_*r*(*j*)_+*ε*_*ijr*,_ where *y*_*ijr*_ is the phenotype value of the *i*th genotype in the *j*th environment and the *r*th block, *μ* is the population mean; *G*_*i*_ is the effect of the *i*th genotype, *E*_*j*_ is the effect of the *j*th environment, (*GE*)_*ij*_ is the genotype-by-environment interaction effect, and *B*_*r*(*j*)_ is the effect of the *r*th replicate block in *j*th environment; and *ε*_*ijr*_ is the random error. The broad-sense heritability values (*h*^*2*^) were estimated as *h*^*2*^ = *σ*^2^_g_/(*σ*^2^_g_+*σ*^2^_ge_/*n*+*σ*^2^/*nr*), where *σ*^2^_g_ is the genetic variance, *σ*^2^_ge_ is the interaction of genotype with environment, *σ*^2^ is the residual error, *n* was the number of environments, and *r* is the number of replications [68]. The best linear unbiased predictor (BLUP) for each genotype across two environments was predicted with the lme4 package in R (http://www.R-project.org/) and used as the phenotypic input in the genome-wide association study (GWAS).

### SNP genotyping and GWAS

The 196 soybean accessions used in this study were genotyped using the NJAU 355K SoySNP Array (S1 File). After excluding SNPs with a MAF < 0.05, 207,608 SNPs were left. The mean values of phenotypic data for all genotypes in the NJ environment and NT environment were separately used to perform the GWAS. Meanwhile, the BLUP values across these two environments were also used to perform the marker-trait association analysis. The GWAS was performed using a compressed mixed linear model (CMLM), which accounted for the complex population structure and familial relatedness [69]. For the CMLM analysis, we used the equation y = W*v*+Xβ+Zu+e, where y is a vector of a phenotype; *v* and β are unknown fixed effects representing marker effects and population structure effects, respectively; and u is a vector for unknown random polygenic effects. W, X and Z are the incidence matrices for *v*, 0 and u, respectively, and e is a vector of random residual effects. All analyses were conducted with an R package called Genome Association and Prediction Integrated Tool (GAPIT) [70]. The population structure was accounted for by a principle component analysis (PCA), and the first five principal components were included in the GWAS model. The kinship matrix was calculated by the VanRaden method [71] and used as the covariance structure of random polygenic effects. The threshold for significant association was set to 1/n (n is the marker numbers, *P* < 4.82×10^−6^) [72].

### Determination of expression levels by quantitative real-time PCR (RT-PCR)

The expression pattern analysis of *GmMYB29* and *IFS2* in response to reduced glutathione (GSH) and insect as well as in different soybean tissues was conducted in NJAU-C041 (Jianliniumaohuang), which was randomly selected from the 196 soybean accessions. The soybean seedlings used for both GSH and insect induction expression analyses were grown in growth chambers under the conditions of 16/8 h (day/night), 28/23°C (day/night), and 70% relative humidity. Soybean Jianliniumaohuang used for the tissue expression analysis was grown under natural conditions in the field at Nanjing Agricultural University.

To analyze the expression of *GmMYB29* and *IFS2* in response to GSH, six pieces of healthy and fully expanded upper-third leaves from different individual 30-day-old soybean plants were excised and immediately submerged in 100 mL of a GSH preparation (10 mM, pH 5.8) containing 0.005% Silwet to reduce leaf surface tension in each beaker flask (250 mL). Leaves from the same location in the control plants were also detached and submerged in a solution (pH 5.8) without GSH. Both control and treated leaves were incubated in an oven-controlled crystal oscillator at 25°C in the dark with gentle shaking (100 rpm). Samples were collected by filtration at four sampling times (0, 3, 6, and 7 h after incubation) [29, 33].

The expression of *GmMYB29* and *IFS2* in response to insects was analyzed by placing two third-instar common cutworm larvae of a uniform size on each fully expanded upper-third leaf of intact 30-day-old soybean seedlings in growth chambers. Control plants were not exposed to common cutworms. The damaged upper-third leaves of treated plants and undamaged leaves at the same location on control plants were excised at eight sampling times (0, 1, 2, 4, 6, 8, 12 and 24 h after induction) for the identification of induced expression [73].

To analyze the expression of *GmMYB29* and *IFS2* in different soybean tissues, RNA samples were isolated from roots, stems, leaves, and flowers during the full-blossom period, pod walls on 10^th^ day after flowering (DAF), seeds at 10, 20, 25, 30, 40 and 50 DAF, and mature seeds.

The expression level of *GmMYB29* was also detected at 40 DAF in the seeds of a subset of 30 soybean accessions, representing varieties with high, medium and low isoflavonoid contents from the 196 accessions.

All collected samples were placed in 2 mL cryopreservation tubes, immediately frozen in liquid nitrogen and stored at -70°C. A total of 100 mg of each sample was used for RNA isolation with the plant RNA Extract Kit (TIANGEN, Beijing, China). cDNA was synthesized using a TaKaRa Primer Script^™^ RT reagent kit with gDNA Eraser according to the manufacturer’s instructions. Gene expression was determined by RT-PCR assays using an ABI 7500 system (Applied Biosystems, Foster City, CA, USA) with SYBR Green Realtime Master Mix (Toyobo). The constitutively expressed gene *Gmtublin* (GenBank accession number: AY907703) was used as a reference gene for RT-PCR. Three replicates were performed for each reaction, and the data were analyzed using the ABI 7500 Sequence Detection System (SDS) software version 1.4.0. The normalized expression, reported as the fold change, was calculated for each sample as ^ΔΔ^CT = (C_T, Target_-C_T, Tubulin_)_genotype_-(C_T, Target_-C_T, Tubulin_)_calibrator_ [74]. The primers used are listed in S4 Table.

### Subcellular localization

*Glycine max* var. Williams 82 was the first whole-genome sequenced soybean with the most complete genome information. To obtain the accurate *GmMYB29* sequence, we cloned it from this cultivar. To determine the subcellular localization of *GmMYB29* in soybean, *GmMYB29* was amplified from the cDNA, including the 5’- and 3’-UTRs, of Williams 82 and cloned into the pAN580 vector containing a GFP expression cassette (pAN580-GFP) to generate the recombinant plasmid pAN580-MYB29-GFP. The recombinant plasmid and the empty control plasmid pAN580-GFP were introduced into onion epidermal cells by gene gun and *Arabidopsis* mesophyll protoplasts by polyethylene glycol (PEG). Transgenic cells were analyzed by a laser scanning confocal microscope using a Zeiss LSM780 camera.

### Transient promoter assays

*GmMYB29* amplified from *Glycine max* var. Williams 82 was inserted into the BamHI-NotI-digested GFP-removed pAN580 vector to generate the effector vector CaMV 35S::MYB29. The open reading frame (ORF) of luciferase (LUC) was cloned from the pGL3 vector (XbaI-XmaI) (Promega, Madison, WI, USA) and introduced into the GFP-loss pAN580 vector to produce the CaMV 35S::LUC plasmid. The CaMV 35S::LUC plasmid was digested by SacI and NheI to remove CaMV 35S, and then the promoter sequence of *IFS2* or *CHS8*, amplified from *Glycine max* var. Williams 82, was inserted to form the reporter vectors IFS2pro::LUC and CHS8pro::LUC. A Renilla luciferase reporter was used as an internal control for normalization. The transient promoter activity in protoplasts derived from *Arabidopsis* suspension cells was analyzed by following the Dual Luciferase Assay protocol (Promega).

### Soybean hairy root transformation

*GmMYB29* was inserted into pBA002 with the CaMV 35S promoter to produce the pBA002-MYB29 overexpression vector. The RNAi vector was constructed using the Gateway technology with a Clonase II Kit (Invitrogen, Carlsbad, CA). A specific 442-bp fragment of the *GmMYB29* cDNA sequence was amplified from Williams 82, and attB1 and attB2 adapters were added. Next, through the BP and LR reactions, we cloned the specific fragment into the pB7GWIWG2(II) vector to obtain the pBI-MYB29Ri vector. As the soybean cultivar Jack is known for its high transformation efficiency, the soybean hairy root transformation was performed using this accession with the pBA002-MYB29 overexpression vector, the pBI-MYB29Ri vector, and the respective empty vectors as controls. The positive hairy roots detected by PCR from several independent transgenic lines were harvested separately and used for gene expression or isoflavone content analysis.

### *GmMYB29*-based association analysis

Using the 5'- and 3'-UTR sequences of *GmMYB29*, which shared relatively low sequence similarity with a paralogous gene, a pair of gene-specific primers (GmMYB29-SF and GmMYB29-SR) were designed (Prime 5.0) to amplify *GmMYB29* from 30 soybean genotypes (S3 and S4 Tables). The primers used to sequence the *GmMYB29* PCR products are listed in S4 Table. The sequences were assembled and aligned using ContigExpress in Vector NTI Advance 10 (Invitrogen, Carlsbad, CA) and MEGA version 6 [75], respectively. Polymorphisms, including SNPs and indels, with a MAF ≥ 0.05 were identified among these genotypes, and their association with isoflavone content and pairwise LDs were calculated by Tassel 5.0 [27, 76]. Markers were defined as being significantly associated with the phenotype on the basis of a significant association threshold (-Log*P* > 1.30, *P* < 0.05).

## Supporting information

S1 Fig Distribution of major isoflavone components of soybean across two environments: Nanjing (NJ) and Nantong (NT). TIC: total isoflavone contents; DAC: daidzein contents; GEC: genistein contents; GLC: glycitein contents.
(TIF)

S2 Fig Alignment of putative phenylpropanoid transcriptional regulators. Multiple alignment of putative R2R3-MYB type transcription factors that regulate isoflavonoid and flavonoid synthesis in various plant species: *Nicotiana tabacum* (NtMYB2), *Arabidopsis thaliana* (AtMYB13, AtMYB14, and AtMYB15), *Daucus carota* (DcMYB1), *Lotus japonicas* (LjMYB12, LjMYB13, LjMYB14, and LjMYB15), *Vitis vinifera* (VvMYB14, VvMYB15, VvMYBF1, VvMYBA2, and VvMYBPA1) and *Glycine max* (GmMYB29, red arrow). Gray boxes below the alignment represent the R2R3-type domain of the MYB factors. Black boxes represent the putative C-terminal SG2 amino acid motif, previously described as the stress response motif [30–32].
(TIF)

S3 Fig Phylogenetic tree of putative phenylpropanoid transcriptional regulators. The phylogenetic tree was constructed using MEGA6 based on the neighbor-joining (NJ) method. The numbers next to the nodes are bootstrap values from 1000 replicates. Predicted functions of the proteins are given beside the tree. Mammalian C-MYB factor was used as an out-group.
(TIF)

S4 Fig Subcellular localization analysis of *GmMYB29*. (A) Expression of 35S::GmMYB29::GFP in onion cells. (B) Expression of 35S::GmMYB29::GFP in *Arabidopsis* mesophyll protoplasts. (C) Expression of 35S::GFP in onion cells. (D) Expression of 35S::GFP in *Arabidopsis* mesophyll protoplasts.
(TIF)

S5 Fig GmMYB29-mediated induction of the activity of *IFS2* promoter constructs with different lengths. (TIF)

S6 Fig Phenotypes of *GmMYB29* transgenic hairy roots and control hairy roots (CK). GmMYB29-OE represents *GmMYB29*-overexpressing roots. GmMYB29-Si represents *GmMYB29*-silenced roots.
(TIF)

S7 Fig Confirmation of transgene-positive soybean hairy roots. (A) The PCR verification of hairy roots overexpressing *GmMYB29* and control hairy roots (CK) was performed using the primers (*35S*-F+*GmMYB29OE*-R) to detect a 688-bp fragment and using the primers (Bar-F+Bar-R) to detect a 482-bp fragment of the phosphinothricin acetyl transferase (*bar*) gene. M, Marker; v, vector positive control; ck, soybean hairy roots transformed with the control vector pBA002; 1 to 9, individual lines transformed with the binary vector pBA002-MYB29. (B) The PCR verification of *GmMYB29*-silenced hairy roots and control hairy roots (CK) was performed using the primers (*35S*-Terminate+*GmMYB29Ri*-R; *GmMYB29Ri*-R+*35S*) to amplify the 609-bp and 623-bp fragments and using the primers (Bar-F+Bar-R) to detect a 482-bp fragment of the *bar* gene. M, Marker; v, vector positive control; ck, soybean hairy roots transformed with the control vector pB7GWIWG2(II); 1 to 9, individual lines transformed with the vector pBI-MYB29Ri.
(TIF)

S8 Fig Relative *GmMYB29* transcript level after overexpression and silencing of *GmMYB29*. (A) Overexpression of *GmMYB29* leads to an increased transcript level in soybean hairy roots. GmMYB29OE1-4 represent four independent *GmMYB29*-overexpressing roots. The data for each of the four independent OE lines or control represent the means of three replicates with error bars indicating SE. (B) Silencing of *GmMYB29* leads to a decreased transcript level in soybean hairy roots. GmMYB29Si1-4 represent four independent *GmMYB29*-silenced roots. The data for each of the four independent Si lines or control represent the means of three replicates with error bars indicating SE. ** significant at the 0.01 probability level; *** significant at the 0.001 probability level.
(TIF)

S9 Fig Quantitative RT-PCR analysis revealed the relative expression levels of genes involved in isoflavone biosynthesis in *GmMYB29*-overexpressing and *GmMYB29*-silenced roots compared to control hairy roots. (A) Relative expression levels of isoflavone biosynthesis-related genes after the overexpression of *GmMYB29*. GmMYB29-OE represents independent *GmMYB29*-overexpressing roots. (B) Relative expression levels of isoflavone biosynthesis-related genes after silencing of *GmMYB29*. GmMYB29Si represents independent *GmMYB29*-silenced roots. The expression level in the control plant is set to 1. Error bars indicates SE of three independent Si and OE lines. * significant at the 0.05 probability level; ** significant at the 0.01 probability level; *** significant at the 0.001 probability level.
(TIF)

S10 Fig A strong regional linkage disequilibrium was observed on chromosome 20 from 43.28 to 43.51 Mb. (TIF)

S1 Table The significant SNP list for major isoflavone components identified only in a specific environment or the BLUP data set. (XLS)

S2 Table The average isoflavone content and relative *GmMYB29* expression level in seeds from 30 soybean accessions. (XLS)

S3 Table Summary of 196 soybean accessions. (XLS)

S4 Table Primers used in this study. (XLS)

S1 File Genotyping data of 196 accessions. (RAR)
