# Supplementary figures and images for: An R2R3-type MYB transcription factor, GmMYB29, regulates isoflavone biosynthesis in soybean
Source: PLoS Genet. 2017 May 10;13(5):e1006770. doi: 10.1371/journal.pgen.1006770 (PMC5443545; doi:10.1371/journal.pgen.1006770)

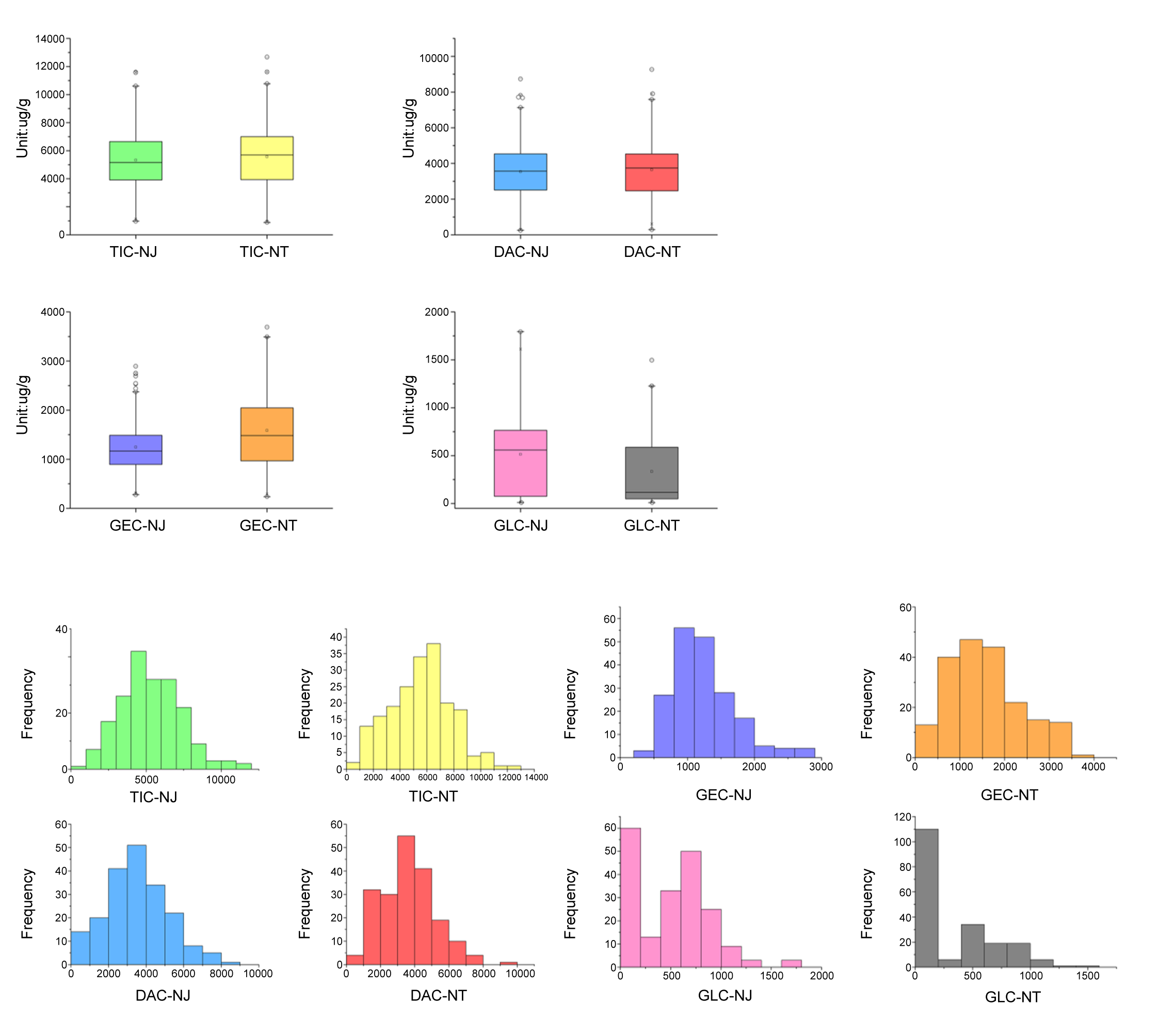

Supplement: S1 Fig — TIC: total isoflavone contents; DAC: daidzein contents; GEC: genistein contents; GLC: glycitein contents. (TIF) [file pgen.1006770.s001.tif]

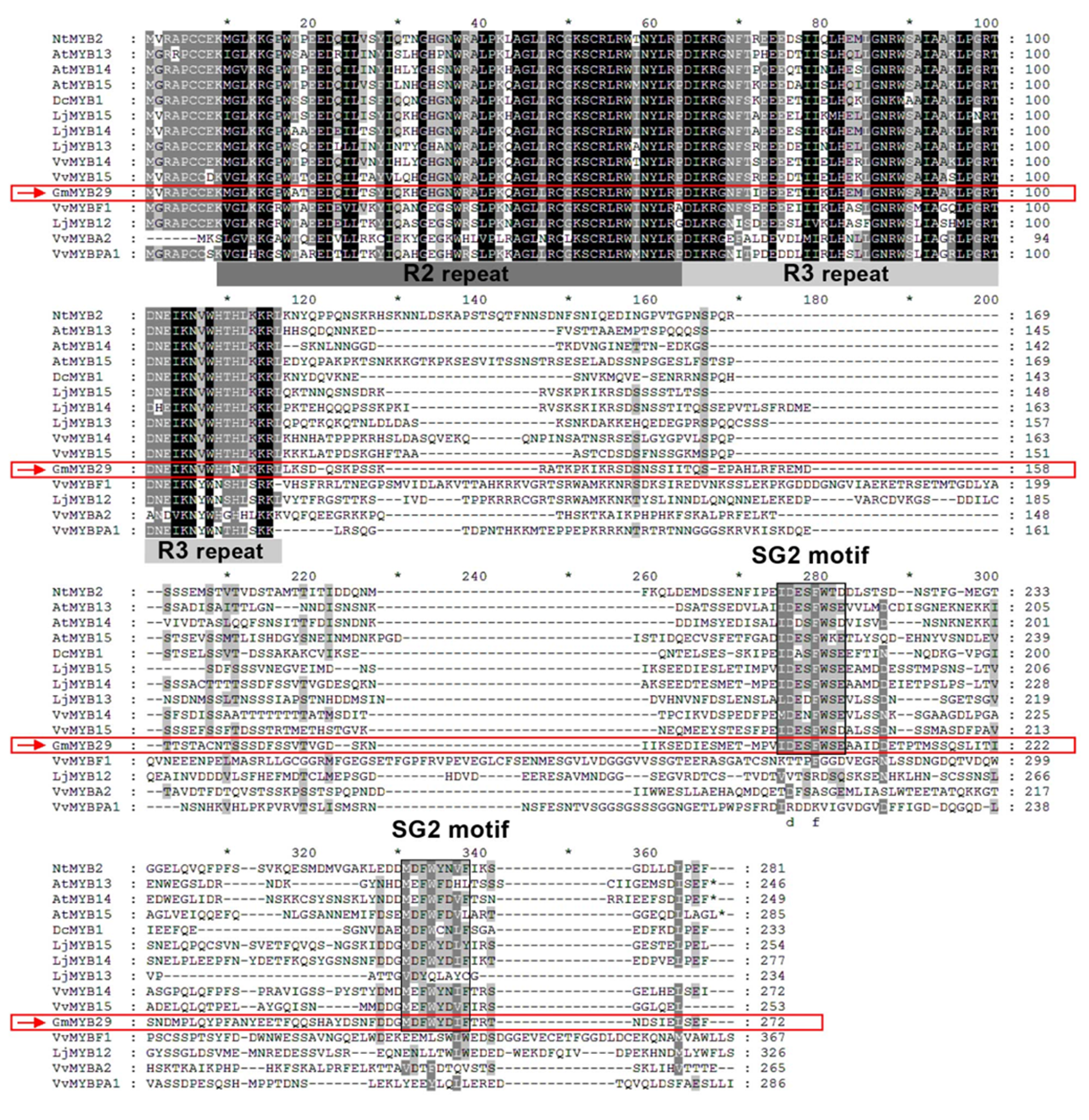

Supplement: S2 Fig — Multiple alignment of putative R2R3-MYB type transcription factors that regulate isoflavonoid and flavonoid synthesis in various plant species: Nicotiana tabacum (NtMYB2), Arabidopsis thaliana (AtMYB13, AtMYB14, and AtMYB15), Daucus carota (DcMYB1), Lotus japonicas (LjMYB12, LjMYB13, LjMYB14, and LjMYB15), Vitis vinifera (VvMYB14, VvMYB15, VvMYBF1, VvMYBA2, and VvMYBPA1) and Glycine max (GmMYB29, red arrow). Gray boxes below the alignment represent the R2R3-type domain of the MYB factors. Black boxes represent the putative C-terminal SG2 amino acid motif, previously described as the stress response motif [30–32]. (TIF) [file pgen.1006770.s002.tif]

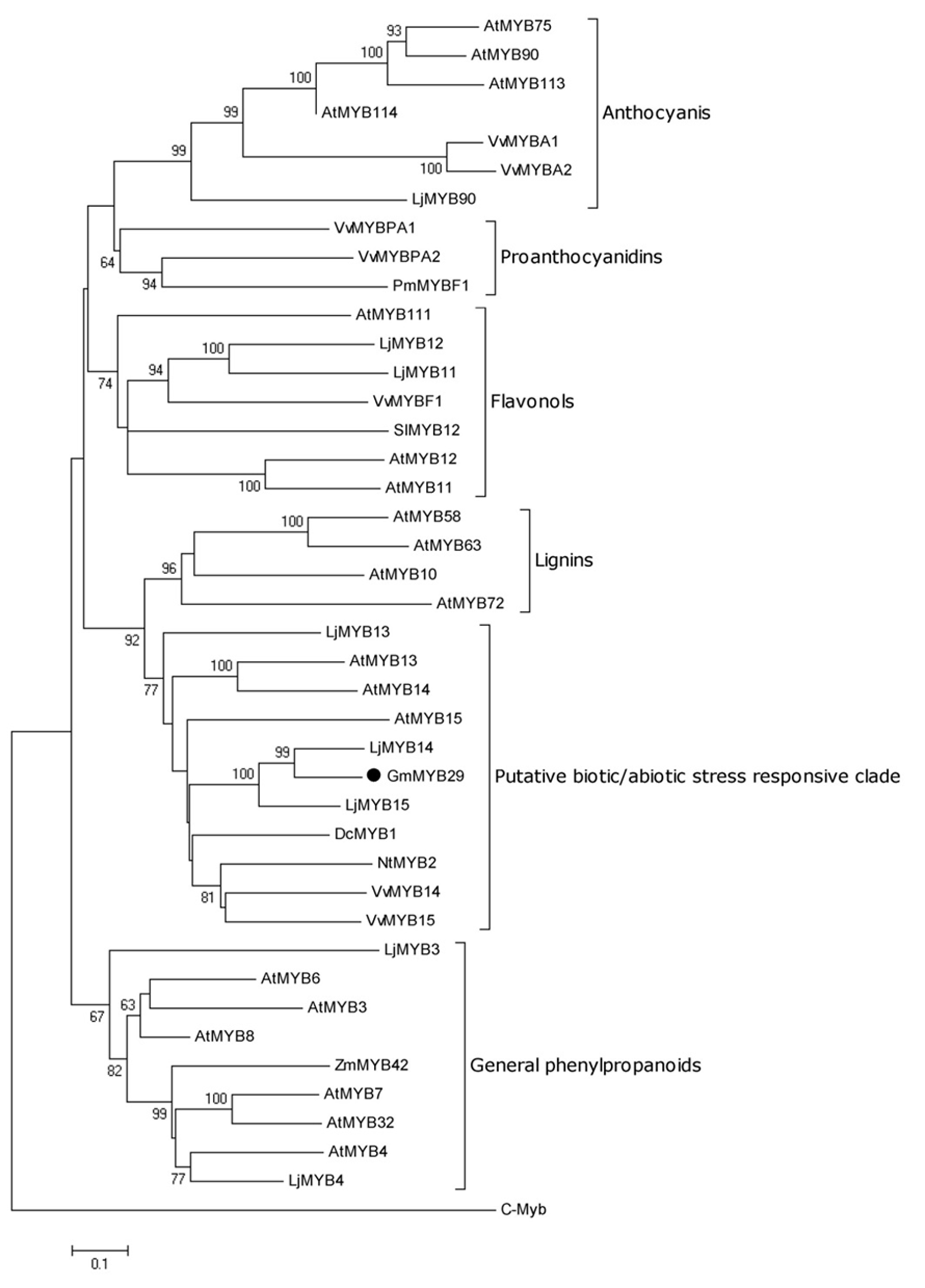

Supplement: S3 Fig — The phylogenetic tree was constructed using MEGA6 based on the neighbor-joining (NJ) method. The numbers next to the nodes are bootstrap values from 1000 replicates. Predicted functions of the proteins are given beside the tree. Mammalian C-MYB factor was used as an out-group. (TIF) [file pgen.1006770.s003.tif]

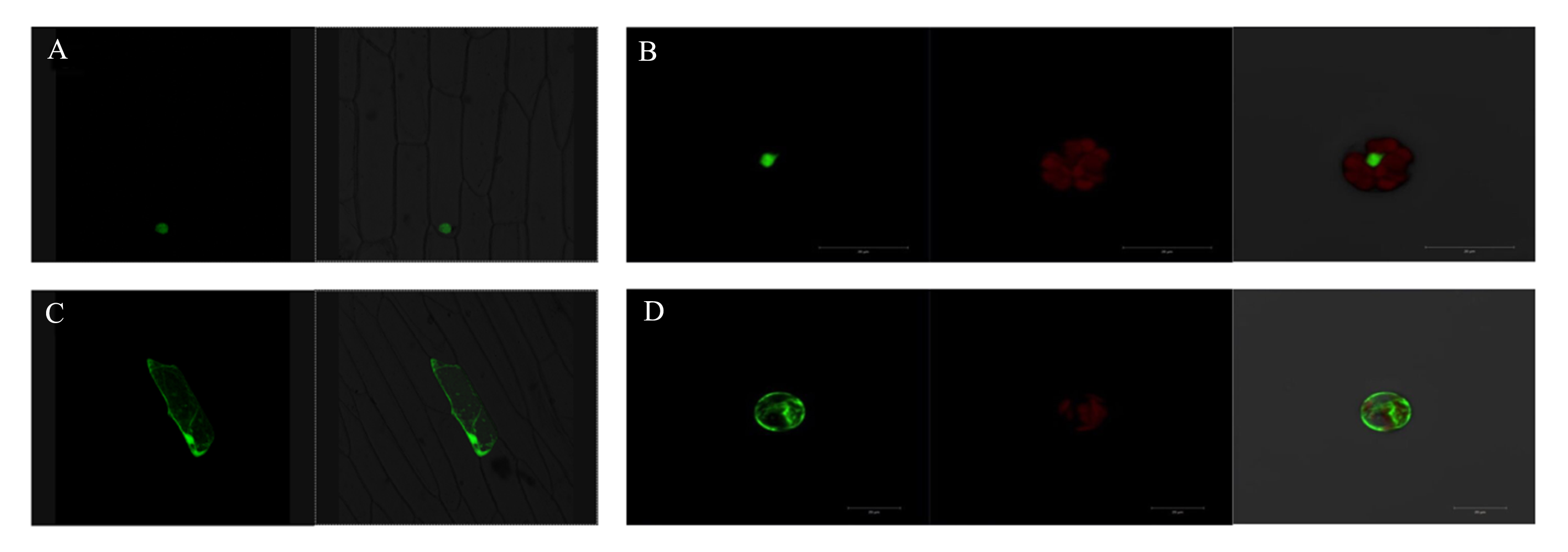

Supplement: S4 Fig — (A) Expression of 35S::GmMYB29::GFP in onion cells. (B) Expression of 35S::GmMYB29::GFP in Arabidopsis mesophyll protoplasts. (C) Expression of 35S::GFP in onion cells. (D) Expression of 35S::GFP in Arabidopsis mesophyll protoplasts. (TIF) [file pgen.1006770.s004.tif]

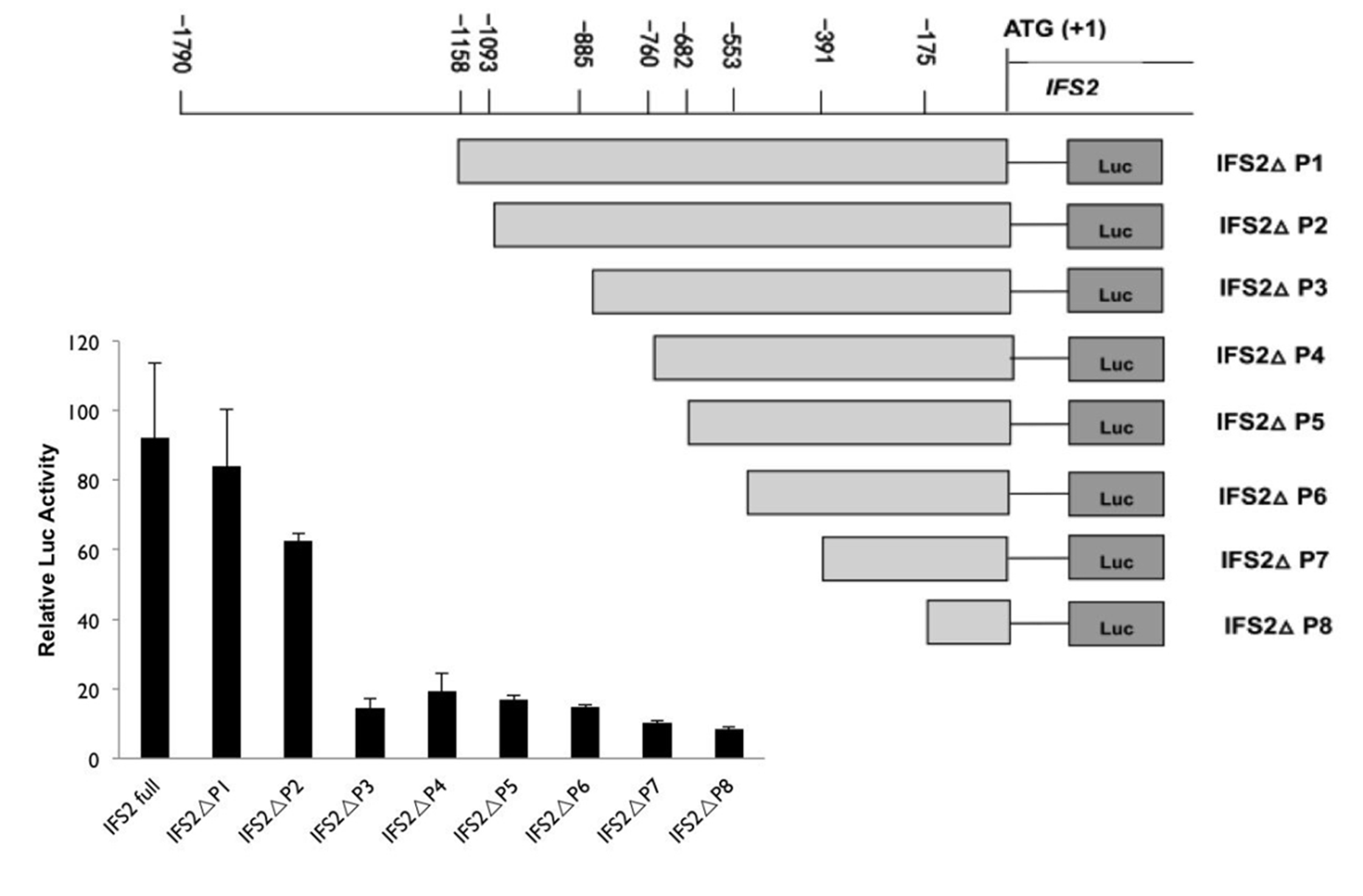

Supplement: S5 Fig — (TIF) [file pgen.1006770.s005.tif]

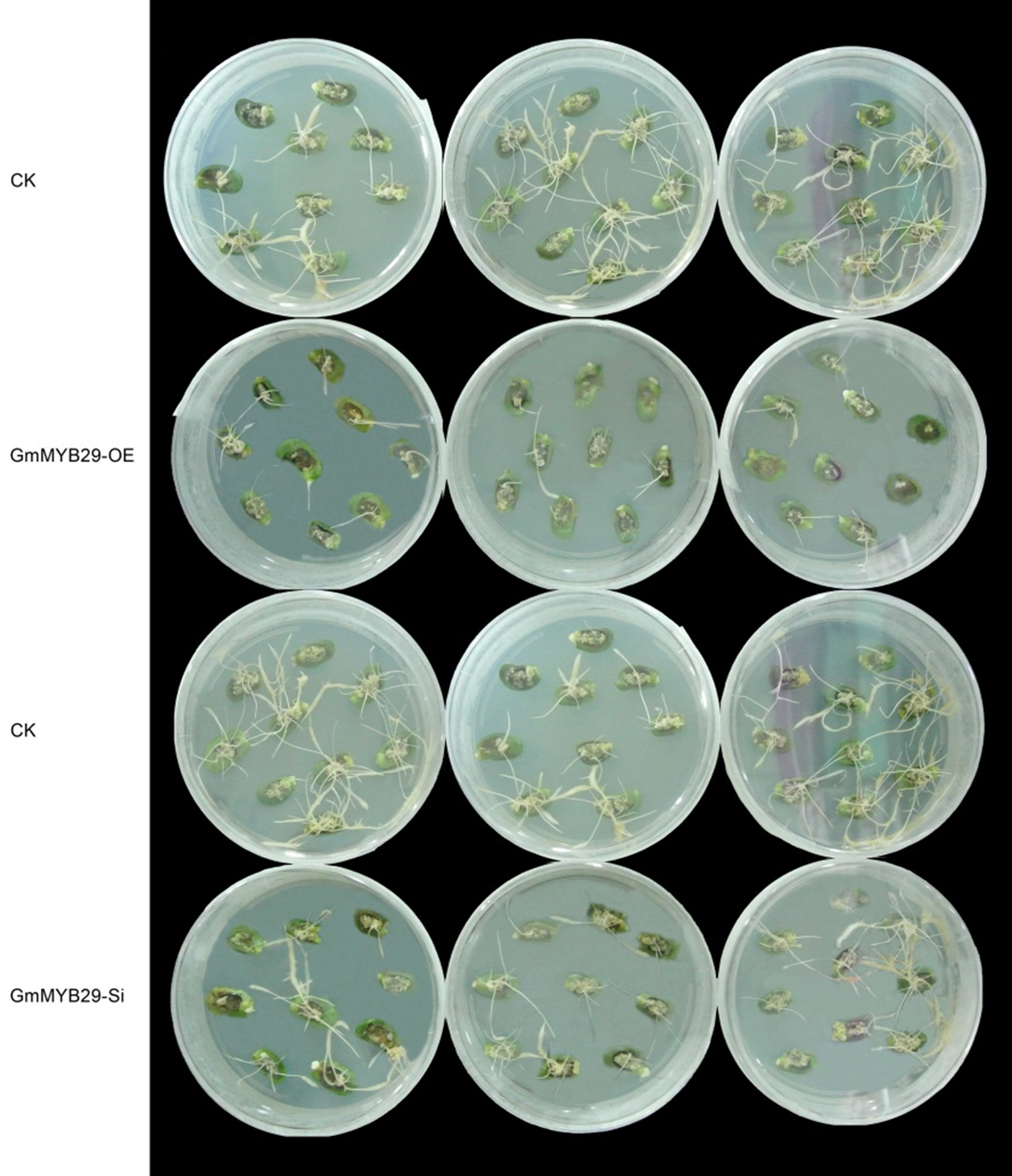

Supplement: S6 Fig — GmMYB29-OE represents GmMYB29-overexpressing roots. GmMYB29-Si represents GmMYB29-silenced roots. (TIF) [file pgen.1006770.s006.tif]

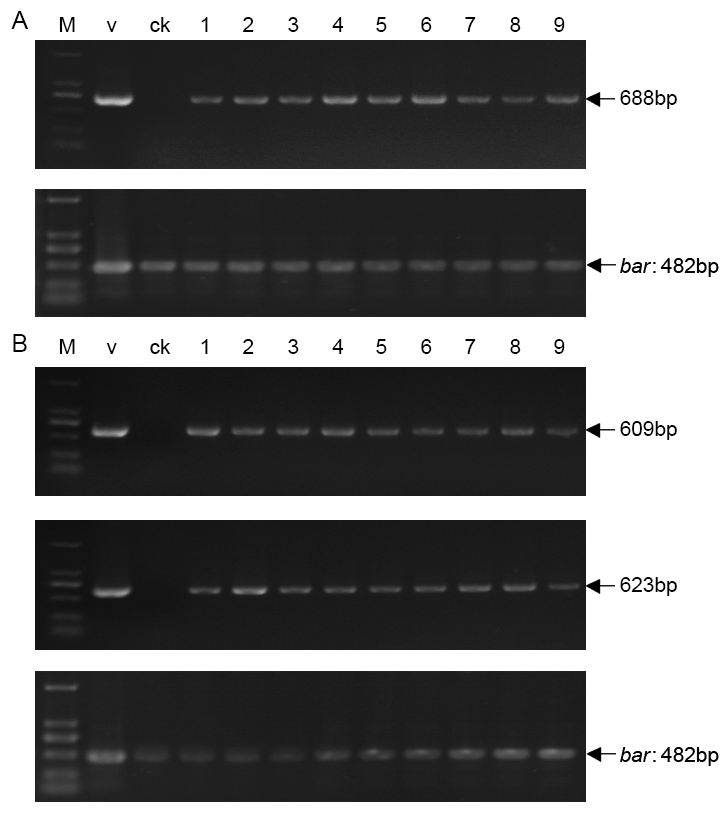

Supplement: S7 Fig — (A) The PCR verification of hairy roots overexpressing GmMYB29 and control hairy roots (CK) was performed using the primers (35S-F+GmMYB29OE-R) to detect a 688-bp fragment and using the primers (Bar-F+Bar-R) to detect a 482-bp fragment of the phosphinothricin acetyl transferase (bar) gene. M, Marker; v, vector positive control; ck, soybean hairy roots transformed with the control vector pBA002; 1 to 9, individual lines transformed with the binary vector pBA002-MYB29. (B) The PCR verification of GmMYB29-silenced hairy roots and control hairy roots (CK) was performed using the primers (35S-Terminate+GmMYB29Ri-R; GmMYB29Ri-R+35S) to amplify the 609-bp and 623-bp fragments and using the primers (Bar-F+Bar-R) to detect a 482-bp fragment of the bar gene. M, Marker; v, vector positive control; ck, soybean hairy roots transformed with the control vector pB7GWIWG2(II); 1 to 9, individual lines transformed with the vector pBI-MYB29Ri. (TIF) [file pgen.1006770.s007.tif]

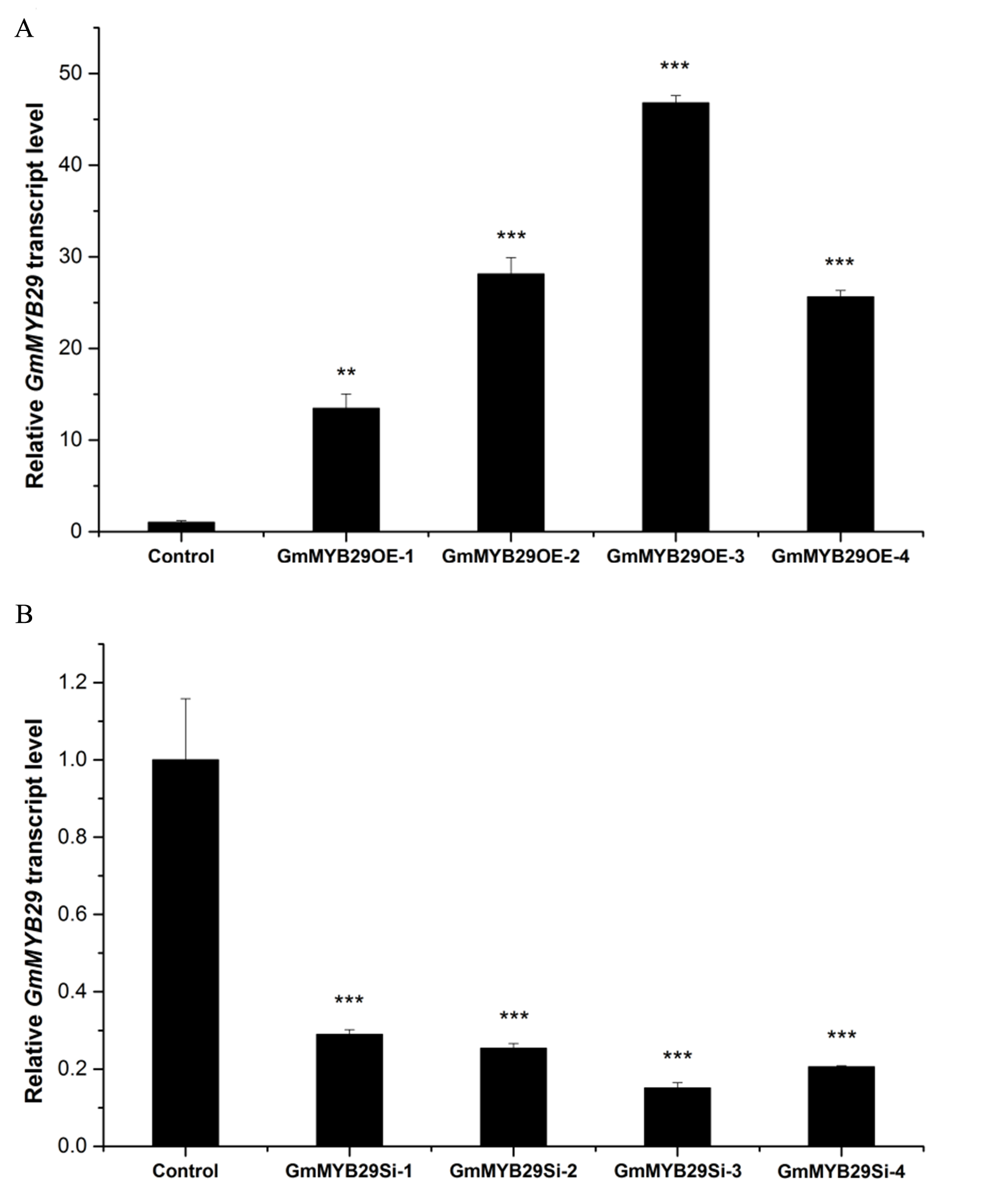

Supplement: S8 Fig — (A) Overexpression of GmMYB29 leads to an increased transcript level in soybean hairy roots. GmMYB29OE1-4 represent four independent GmMYB29-overexpressing roots. The data for each of the four independent OE lines or control represent the means of three replicates with error bars indicating SE. (B) Silencing of GmMYB29 leads to a decreased transcript level in soybean hairy roots. GmMYB29Si1-4 represent four independent GmMYB29-silenced roots. The data for each of the four independent Si lines or control represent the means of three replicates with error bars indicating SE. ** significant at the 0.01 probability level; *** significant at the 0.001 probability level. (TIF) [file pgen.1006770.s008.tif]

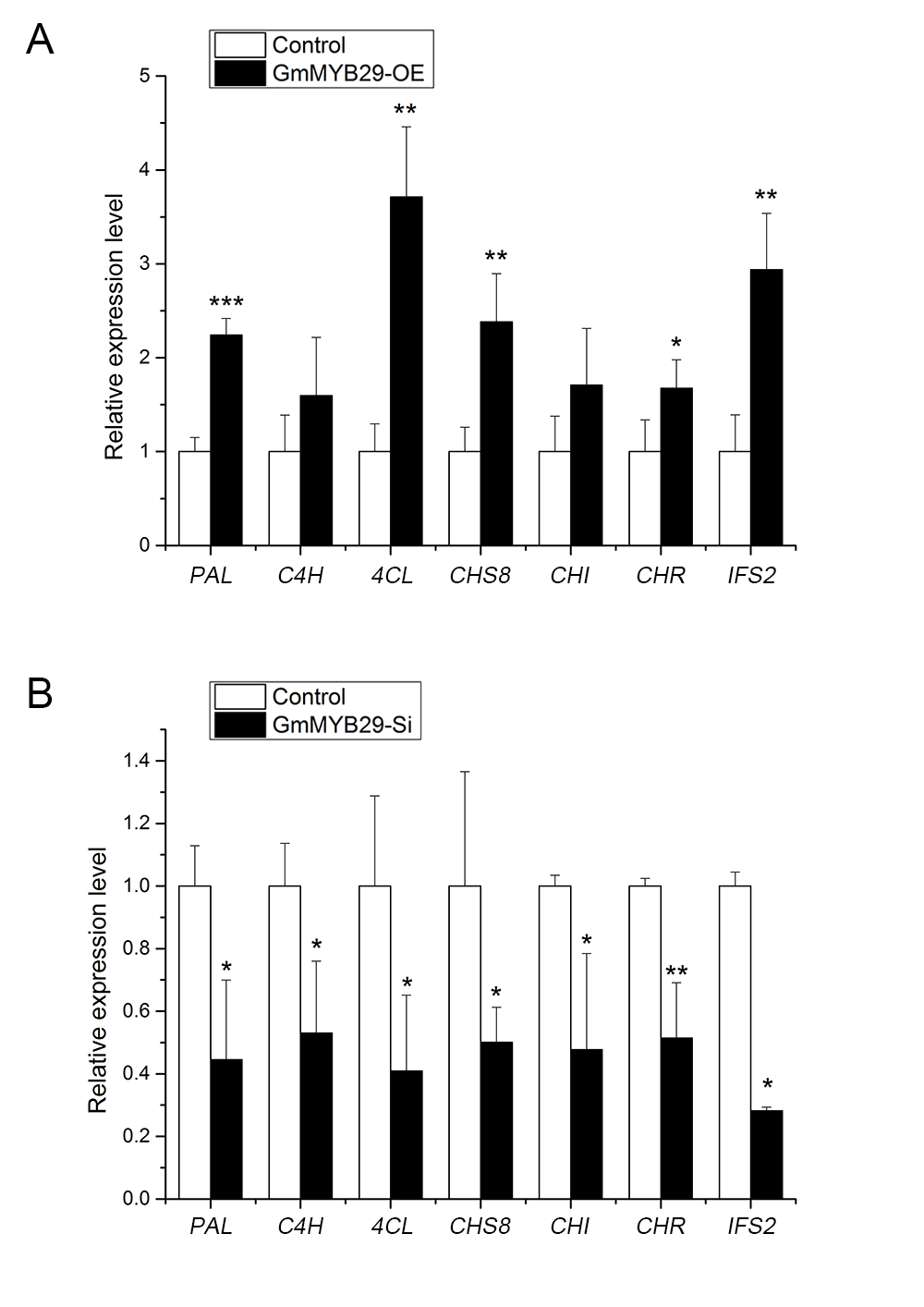

Supplement: S9 Fig — (A) Relative expression levels of isoflavone biosynthesis-related genes after the overexpression of GmMYB29. GmMYB29-OE represents independent GmMYB29-overexpressing roots. (B) Relative expression levels of isoflavone biosynthesis-related genes after silencing of GmMYB29. GmMYB29Si represents independent GmMYB29-silenced roots. The expression level in the control plant is set to 1. Error bars indicates SE of three independent Si and OE lines. * significant at the 0.05 probability level; ** significant at the 0.01 probability level; *** significant at the 0.001 probability level. (TIF) [file pgen.1006770.s009.tif]

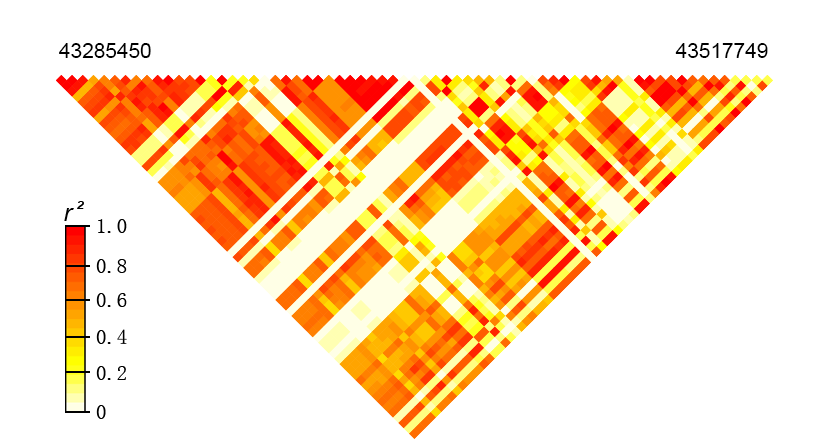

Supplement: S10 Fig — (TIF) [file pgen.1006770.s010.tif]
